# The Early Expansion and Evolutionary Dynamics of POU Class Genes

**DOI:** 10.1093/molbev/msu243

**Published:** 2014-09-25

**Authors:** David A. Gold, Ruth D. Gates, David K. Jacobs

**Affiliations:** ^1^Department of Ecology and Evolution, University of California, Los Angeles

**Keywords:** POU, Metazoa, homeobox, EvoDevo, stem cells, gene duplication

## Abstract

The POU genes represent a diverse class of animal-specific transcription factors that play important roles in neurogenesis, pluripotency, and cell-type specification. Although previous attempts have been made to reconstruct the evolution of the POU class, these studies have been limited by a small number of representative taxa, and a lack of sequences from basally branching organisms. In this study, we performed comparative analyses on available genomes and sequences recovered through “gene fishing” to better resolve the topology of the POU gene tree. We then used ancestral state reconstruction to map the most likely changes in amino acid evolution for the conserved domains. Our work suggests that four of the six POU families evolved before the last common ancestor of living animals—doubling previous estimates—and were followed by extensive clade-specific gene loss. Amino acid changes are distributed unequally across the gene tree, consistent with a neofunctionalization model of protein evolution. We consider our results in the context of early animal evolution, and the role of *POU5* genes in maintaining stem cell pluripotency.

## Introduction

**Fig. 1.**
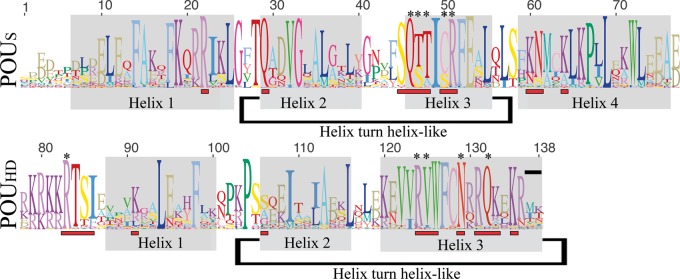
Structure and variation within the POU_S_ and POU_HD_ domains. Probable α-helix subdomains are shaded in gray, amino acids known to make contact with DNA in at least one POU class are marked with a red bar, and the subset which makes direct contacts with nucleotides is marked with an asterisk (based on Klemm et al. 1994; Jacobson et al. 1997; Reményi et al. 2001, 2003; Jauch et al. 2010; Esch et al. 2013). The combined height of the amino acids at each position indicates the degree of sequence conservation, whereas the height of each individual amino acid indicates its relative frequency. This figure is based on the alignment we used for our phylogenetic analyses (see Materials and Methods and supplementary file S1, Supplementary Material online), and was created using the Sequence Logo function in Geneious.

The POU genes represent a large class of DNA-binding transcription factors known for their roles in cell-type specification and developmental regulation (Ryan and Rosenfeld 1997; Phillips and Luisi 2000). The POU homolog *Oct-4* has been extensively studied, as it is the most critical of the four “Yamanaka factors” used to induce pluripotent stem cells in mammals (Niwa et al. 2000; Takahashi and Yamanaka 2006; Ng and Surani 2011). The POU name is an acronym derived from the mammalian genes *Pit-1*, *Oct-1*, and *Oct-2*, as well as the *Caenorhabditis elegans* gene *unc-86*, which all share a 150-amino acid region of high sequence similarity (Herr et al. 1988). Although POU genes have been identified in animals as diverse as sponges and humans, there exists strong conservation within the major domains (fig. 1). POU genes feature a modular, tripartite structure, consisting of an N-terminal POU-specific domain (POU_S_), a C-terminal homeodomain (POU_HD_), and a linker region of varying length connecting the two. The secondary structure of both POU_S_ and POU_HD_ domains consists of a series of α-helices, which make multiple contacts with DNA through hydrogen bonding with the phosphate backbone or directly to nucleotides (Jacobson et al. 1997; Reményi et al. 2001; Jauch et al. 2010; Esch et al. 2013). In both domains, the third helix serves as the recognition helix, binding to the major groove of DNA and making the majority of direct contacts with nucleotides (Assa-Munt et al. 1993; Dekker et al. 1993; Jacobson et al. 1997). As figure 1 suggests, these contact regions are often, though not always, the most invariant sites within the POU class.

Despite this significant conservation, POU proteins are capable of generating high levels of conformational diversity through complex interactions with DNA and other transcription factors. POU genes form a variety of heterodimers and homodimers that can bind to noncontiguous DNA strands (Voss et al. 1991; Jacobson et al. 1997; Scully et al. 2000; Reményi et al. 2001; Rodda et al. 2005). It is common for POU paralogs to share partially overlapping functions (Erkman et al. 1996; Tichy et al. 2008), and certain POU knockouts can be rescued by a paralog that is not normally expressed in the region (Friedrich et al. 2005). Some POU genes take on multiple isoforms, which oppose each other in regulation, or work together to bind multiple trans factors (Konzak and Moore 1992; Lee and Salvaterra 2002; Theodorou et al. 2009). Even changes in the spacing between the two DNA binding domains can allow the same transcript to act as an activator in one scenario and a repressor in another (Scully et al. 2000). The last two amino acids of the homeodomain may be particularly important in driving dimerization (Reményi et al. 2001), which could be as important for the recognition of *cis*-regulatory modules as the DNA-binding interface (Jauch et al. 2010). Interestingly, this final dipeptide appears to be one of the most variable positions and may be a site involved in functional protein evolution (fig. 1).

Since their initial discovery, more than 1,000 POU sequences have been recovered from across the Metazoa. These are generally organized into six families (*POU1*–*POU6*). Multiple POU families have been described in every annotated animal genome, and many lineages, particularly vertebrates, have multiple paralogs in multiple families. The resultant extensive nomenclature is summarized in table 1. POU genes have only been recovered from Metazoa, suggesting that the POU_S_ domain represents an animal novelty that was incorporated into a more ancient homeodomain containing gene during the early evolution of animals (Degnan et al. 2009). However, the presence of multiple, and often nonoverlapping, POU families in early-branching animal lineages makes rooting the POU gene tree difficult, and has led to conflicting topologies in gene tree reconstruction (Kamm and Schierwater 2007; Larroux et al. 2008; Ryan et al. 2010).

**Table 1.** Division of POU Homologs into the Six Major Classes, Including Common Names.

	Mammalian Homologs	*Drosophila* Homologs	*Caenorhabditis* Homologs
POU1	*POU1F1* (*Pit-1*)	None	None
POU2	*POU2F1* (*Oct-1*), *POU2F2* (*Oct-2*), *POU2F3* (*Oct-11*)	*pdm-1* (*nubbin*; *dPOU-19*; *twain*; *dOct1*)	*Ceh-18*
		*pdm-2* (*miti-mere*; *dOct-2*)	
POU3	*POU3F1* (*Oct-6*; *SCIP*), *POU3F2* (*Oct-7*; *Brn-2*), *POU3F3* (*Oct-8*; *Brn-1*), *POU3F4* (*Oct-9*; *Brn-4*; *DFN3*)	*vvl* (*cf1a*; *drifter*)	*Ceh-6*
POU4	*POU4F1* (*Brn-3a*; *RDC-1*; *Oct-T1*), POU4F2 (*Brn-3b*; *Brn-3.2*), POU4F3 (*Brn-3c*; *Brn-3.1*; *DFNA15*)	*acj6* (*Ipou*)	*Unc-86*
POU5	*POU5F1*(*Oct-3*; *Oct-4*), *POU5F2* (*SPRM-1*), *Pou2/V*	None	None
POU6	*POU6f1* (*Brn-5*; *mPOU*), *POU6f2* (*Emb*; *RPF-1*)	*pdm-3*	None

To better understand the diversity and evolution of POU genes, we adopted a comparative genomic approach to reconstruct the class topology. The results of this study were corroborated with gene fishing, using degenerate polymerase chain reaction (PCR) primers to capture novel POU homologs from a variety of understudied animal clades. Finally, we used ancestral state reconstruction to track the most likely trajectory of POU sequence evolution. Taken together, our results suggest that four of the six major families of POU genes (*POU6*, *POU1*, *POU3*, and *POU4*) were present before the last common ancestor of all living animals, which is double the previous estimate. The POU families appear to have evolved primarily through gene duplication followed by neofunctionalization (Lynch and Conery 2000; Innan and Kondrashov 2010), where one paralog retains the ancestral amino acid sequence (and presumably aspects of the ancestral function), whereas the other duplicate incorporates significantly more nonsynonymous mutations.

## Results

### A Comparative Genomics Approach Resolves Many Aspects of the POU Gene Tree Topology

**Fig. 2.**
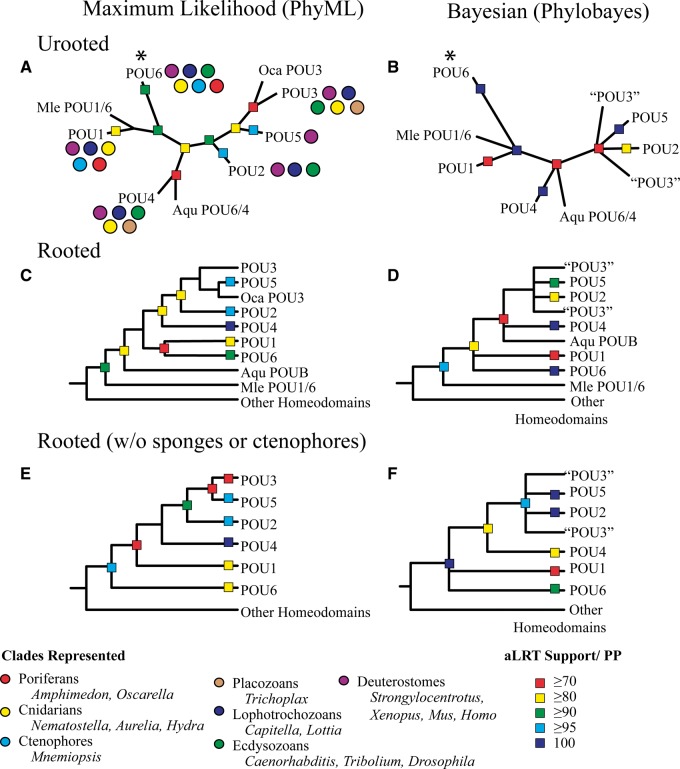
Summary of ML and Bayesian reconstructions of our POU data set. See the Materials and Methods section and supplementary material, Supplementary Material online, for more complete information on taxon sampling and support values for all nodes. Genes with uncertain phylogenetic position from *Amphimedon* (Aqu), *Oscarella* (Oca), and *Mnemiopsis* (Mle) are singled out. (*A*, *B*) Unrooted topologies. The location of the midpoint root is marked with an asterisk. (*C*, *D*) Topologies that have been rooted by the inclusion of additional homeodomains. (*E*, *F*) Rooted topologies with all sequences from *Amphimedon*, *Oscarella*, and *Mnemiopsis* removed.

We began by surveying available animal genomes for POU-domain sequences. For phylogenetic analyses, we ultimately chose a subsample of taxa that included model laboratory animals as well as representatives of major clades from across the animal tree (discussed in detail in the Materials and Methods section). We employed both maximum likelihood (ML) and Bayesian approaches to tree building. To account for the low phylogenetic support of sequences from the sponges *Amphimedon queenslandica* and *Oscarella carmela*, as well as the ctenophore *Mnemiopsis leidyi,* we also attempted tree reconstruction excluding these taxa. The results of these analyses are summarized in figure 2 (see supplementary figs. S1–S6, Supplementary Material online, for full trees).

Although we were unable to generate a single topology across all analyses, we were able to resolve some areas of uncertainty regarding the relationships between POU families. Previous studies rooting the POU class with homeodomains have recovered *POU6* as sister to all other POU families. However, there has been disagreement whether the next family to diverge was *POU4* (e.g., Ryan et al. 2010) or *POU1* (e.g., Larroux et al. 2008; Millane et al. 2011); different rooting methods have also produced alternate topologies for the same data set (Kamm and Schierwater 2007). In contrast, all of our analyses identified *POU1* as the closest paralog to *POU6*, although the nature of that relationship varied, with *POU1* and *POU6* occasionally forming a sister clade or a polytomy (fig. 2*C*, *D*, and *F*). Still, there are several reasons to prefer *POU6* as the outgroup to the other extant POU families. Topologies illustrated in figure 2*C* and *D* include highly divergent and poorly supported sequences from *Mnemiopsis* and *Amphimedon*, which increases the probability of long-branch attraction artifacts. When these sequences are removed, ML strongly supports *POU6* as the outgroup (fig. 2*E*), whereas the Bayesian phylogeny generates a polytomy between *POU6* and *POU1* (fig. 2*F*). Given the small number of phylogenetically informative sites in our alignment, it is likely that the Bayesian approach lacks sufficient information to produce a topology with strong posterior support. Midpoint rooting on the unrooted topologies places the root within the *POU6* family (fig. 2*A* and *B*), and an additional rooting process used during ancestral state reconstruction (discussed in detail in the Materials and Methods section) also supports *POU6* as the outgroup.

Following *POU6* and *POU1*, all of our analyses support *POU4* as the next paralog to diverge. One gene from the sponge *Amphimedon*, which has previously been described as *POUB* (Larroux et al. 2008), has an affinity with *POU4* in some of our analyses, and *POU6* in others. This was followed by either a split between *POU2* and *POU3/5* (maximum-likelihood analyses; fig. 2*A*, *C*, and *E*) or a polytomous *POU3* “bush,” which includes monophyletic *POU2* and *POU5* classes (Bayesian analyses; fig. 2*B*, *D*, and *F*). As figure 2*A* illustrates, *POU3* includes representatives from a number of basally branching animal taxa, including cnidarians, the placozoan *Trichoplax adherans*, and possibly the sponge *Oscarella*. *POU2* was only recovered from bilaterian animals, whereas *POU5* appears restricted to vertebrates, which supports the hypothesis that these families are products of more recent, clade-specific duplications.

### Gene Fishing Recovers Putative POU3 and POU4 Classes in Sponges

By using a diverse selection of taxa, we uncovered several unanticipated results regarding the distribution of POU genes across the animals. First, our analyses provide good support for a *POU3* homolog in *Oscarella*, as well as moderate support for a *POU4* homolog in *Amphimedon*. This potentially doubles the number of POU families identified in the sponges, as previous analyses have only recognized *POU6* and *POU1* homologs in the Porifera (Larroux et al. 2008). A second surprise comes from the taxon distribution of the *POU1* family. *POU1* is present in early-branching animals, such as cnidarians, ctenophores, and sponges, as well as vertebrates and the chordate amphioxus (Jacobs and Gates 2003; Candiani et al. 2008). Our analyses suggest that *POU1* is also present in the annelid *Capitella teleta*, but absent from all other sampled protostomes. The identification of *POU1* in annelids is not new, as it has previously been described in the polychaete worm *Platynereis dumerilii* (Raible et al. 2005), but the hypothesis that the annelids are the only protostomes to retain this homolog has not been formalized. Indeed, although we were also able to recover a candidate *POU1* from the genome of the leech *Helobdella robusta* (supplementary fig. S7, Supplementary Material online), we found no other protostome *POU1* candidates in the NCBI database, or in any additional publically available protostome genomes.

To corroborate these results, we performed a gene fishing experiment, using degenerate PCR primers to amplify POU genes from a variety of understudied animal lineages (summarized in table 2). Family designations for the recovered genes were determined using Basic Local Alignment Search Tool (BLAST), alignments of the linker regions (supplementary fig. S7, Supplementary Material online), and phylogenetic analysis (supplementary fig. S8, Supplementary Material online). In our phylogenetic analyses, the linker was discarded, for although the region is often conserved within POU families, it is difficult to homologize between them. However, this also makes the linker a good candidate for supporting family affinity, as it reduces the probability that our phylogenetic results are caused by convergent evolution within the otherwise largely invariant POU_S_ and POU_HD_ domains.

**Table 2.** Results of Gene Fishing Experiments.

Species Name	Phylum	Class	POU Genes Recovered
*Acarnus erithacus*	Porifera	Demospongiae	*POU1*, *POU4* (2)
*Tethya aurantia*	Porifera	Demospongiae	*POU4*
*Spongilla* sp.	Porifera	Demospongiae	*POU4*
*Haliclona* sp.	Porifera	Demospongiae	*POU1*
*Rhabdocalyptus dawsoni*	Porifera	Hexactinellida	*POU1* (2), *POU3*
*Pleurobrachia* sp.	Ctenophora	Tentaculata	*POU1*
*Agaricia* sp.	Cnidaria	Anthozoa	*POU1*, *POU3*
*Anthopleura elegantissima*	Cnidaria	Anthozoa	*POU3*
*Fungia* sp.	Cnidaria	Anthozoa	*POU1*
*Pelagia colorata*	Cnidaria	Scyphozoa	*POU4*
*Convolutriloba* sp.	Acoelomorpha	Acoela	*POU3*, *POU4*
*Notoplana acticola*	Platyhelminthes	Turbellaria	*POU3*, *POU4*
*Stylochus tripartitus*	Platyhelminthes	Turbellaria	*POU3*, *POU4* (3)
*Alitta virens* (formally *Nereis virens*)	Annelida	Polychaeta	*POU3*, *POU4*
*Phragmatopoma californica*	Annelida	Polychaeta	*POU4*
*Hydroides* sp.	Annelida	Polychaeta	*POU4*
*Acanthina* sp.	Mollusca	Gastropoda	*POU4*
*Kelletia kelletii*	Mollusca	Gastropoda	*POU3* (2), *POU4*
*Transennella* sp.	Mollusca	Bivalvia	*POU4*
*Crassostrea gigas*	Mollusca	Bivalvia	*POU3*

Our gene fishing results are consistent with comparative genomic inferences regarding clade-specific gene gain and loss of POU families. The recovery of a *POU3* homolog in the hexactinellid *Rhabdocalyptus dawsoni* and *POU4* homologs from the demosponges *Acarnus erithacus*, *Tethya aurantia*, and *Spongilla* sp. strongly supports our interpretation of the *Amphimedon* and *Oscarella* data, and collectively doubles the number of POU families known from the sponges. Because sponges commonly house a variety of symbiotic and commensal organisms (Brusca RC and Brusca GJ 2003), contamination is a concern. However, a number of observations argue against contamination. First, we obtained different *POU* genes from different sponge clades, with *POU4* being exclusive to demosponges (including *Amphimedon*), whereas *POU3* was recovered in the hexactenellid *Rhabdocalyptus* and the homoscleromorph *Oscarella*. Second, we obtained *POU4* genes from both the saltwater demosponges *Acarnus* and *Thethya*, as well as the freshwater sponge *Spongilla*. Third, although the sponge *POU* genes do not form monophyletic clades in phylogenetic analyses (supplementary fig. S8, Supplementary Material online), they also show no consistent affinity to any other animal phyla across NCBI BLAST searches. Consequently, we infer that *POU1*, *POU3*, *POU4*, and *POU6* were all present in the last common ancestor of sponges.

As with any gene fishing expedition, one must be cautious about making hard conclusions regarding gene absence. For example, we did not recover any *POU2* or *POU6* genes, even though *POU6* homologs have been identified in every annotated metazoan genome (excluding nematodes) and *POU2* in every annotated bilaterian genome. Therefore, it is unclear how we should interpret our failure to recover *POU1* genes from the annelids *Alitta, Phragmatopoma,* or *Hydroides*, despite their presence in the three annelid genomes. A previous gene fishing study also failed to recover *POU1* in the earthworm *Lumbricus terrestris* (Shah et al. 2000), which suggests that *POU1* has either been lost in many annelid lineages, or that annelid *POU1* genes are difficult to amplify with degenerate primers.

Still, the distribution of gene absences might provide some information. For example, our inability to find *POU3* or *POU4* homologs in *Pleurobrachia* supports the hypothesis that these families are absent from the two major ctenophore lineages (the Tentaculata and Nuda), and thus missing from the phylum altogether. Similarly, although *POU1* genes were recovered from cnidarians, sponges, and a ctenophore, none was recovered from flatworms, acoels, or molluscs, which is consistent with their absence in publically available genomes.

### Ancestral State Reconstruction Supports a Pattern of Gene Duplication Followed by Protein Neofunctionalization

**Fig. 3.**
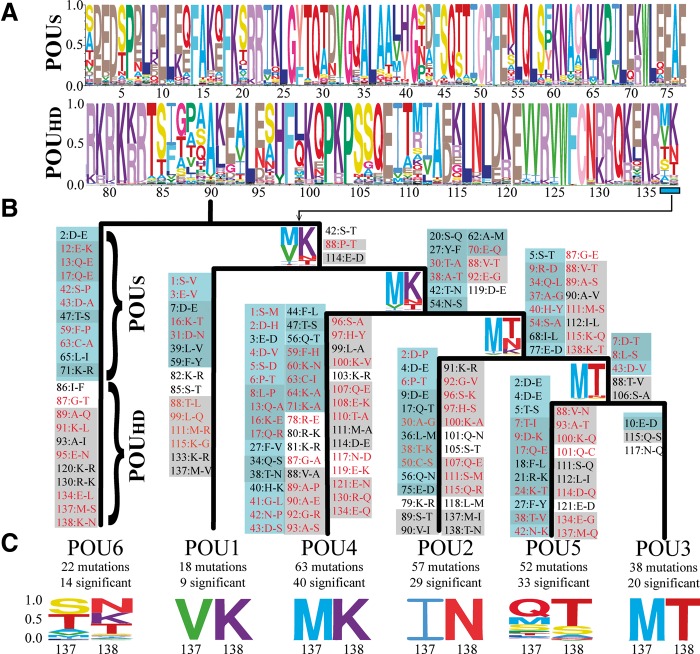
Ancestral sequence and evolutionary trajectory of the POU_S_ and POU_HD_ domains. (*A*) Ancestral state reconstruction of the original POU_S_ and POU_HD_ domains. The probability of an amino acid being the ancestral state at each site is represented by the height of the letter, with the most probable peptide at the top. (*B*) Amino acid substitutions that occurred at each node, based on the most likely peptide at each node versus the ancestral node. Significant amino acid substitutions (i.e., moving between amino acids with positively charge, negatively charge, polar uncharged, or hydrophobic side chains) are colored in red. Mutations in the POU_S_ domain are highlighted in blue, and mutations occurring within an α-helix subdomain are highlighted in gray. (*C*) Total number of mutations that occurred between the common ancestor of each POU class and the ancestral POU sequence. The probability of the final dipeptide for the ancestor of each POU class is visualized at the bottom of the figure, and at the bifurcation of each ancestral node.

Resolving the topology and affinity of metazoan POU homologs allowed us to study the directionality of evolution within the POU_S_ and POU_HD_ domains. We used maximum-likelihood-based ancestral state reconstruction on a species-tree-corrected gene tree to track all amino acid changes that occurred at each node up to the common ancestor of the extant POU classes (fig. 3). Out of 173 amino acid changes, 117 occurred within an α-helix domain, and 95 changes were “significant,” which we define as a shift from one major type of amino acid to another (i.e., positively charged [K, R, H], negatively charged [D, E], hydrophilic [S, T, N, Q, C, G, P], and hydrophobic [A, I, L, M, F, W, V, Y]). Our results suggest that mutations are not distributed evenly across the tree; after most bifurcations, one lineage appears to accumulate more amino acid changes than the other. To verify this pattern, we used the DIVERGE (v3.0) software package to perform pairwise comparisons between gene families (Gu et al. 2013; see Materials and Methods for more information). In our tests for differences in significant amino acid substitution rates, we determined that *POU4* was significantly different from *POU2*, *POU3*, or *POU5*, and that *POU5* was significantly different from *POU2*. Taken collectively, our analyses suggest three major times of significant increase in amino acid substitutions 1) When *POU6* split from the last common ancestor of all other POU families, 2) when *POU4* split from the last common ancestor of *POU2/3/5*, and 3) when *POU5* and *POU2* split from *POU3*. These results appear consistent with neofunctionalization models of gene duplication, which predict purifying selection on one gene duplicate, and a release of purifying selection combined with the evolution of a new adaptive function in the other duplicate (Innan and Kondrashov 2010). This would be in contrast to subfunctionalization models that predict relaxed purifying selection on both gene duplicates (Innan and Kondrashov 2010). These results also lead to some unintuitive conclusions regarding the similarity between extant POU families and their ancestral nodes. For example, although *POU6* appears to be the earliest branching family, *POU1* has accumulated far fewer significant amino acid substitutions during its evolution, presumably as a function of purifying selection and functional continuity. Similarly, although *POU2* appears to be sister to a *POU3*/*POU5* clade, *POU3* has accumulated far fewer significant substitutions since splitting from the common ancestor than either *POU2* or *POU5*. Similar to our presence–absence data described earlier, these results are consistent with the hypothesis that the last common ancestor of the *POU2/3/5* superfamily was *POU3*-like, and that *POU2* and *POU5* represent clade-specific duplications in bilaterians and vertebrates, respectively.

**Fig. 4.**
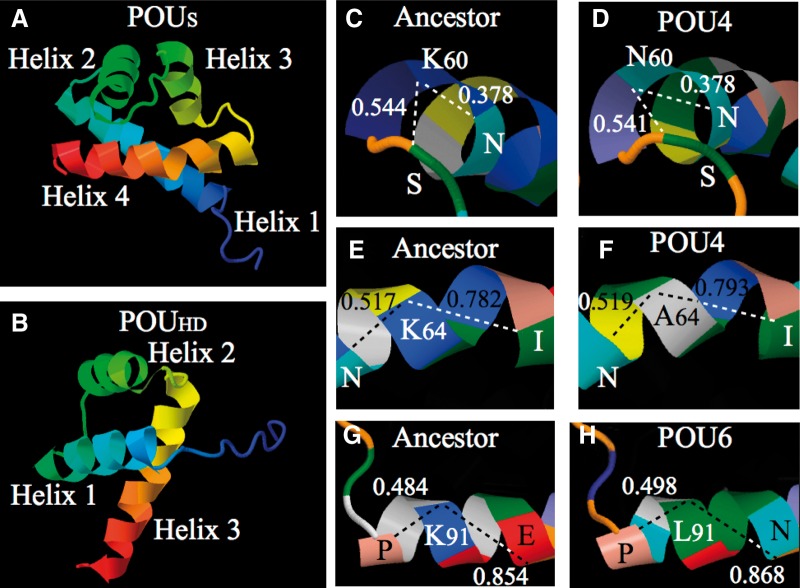
Predicted structure of the ancestral POU_S_ and POU_HD_ domains, and the effects of significant amino acid substitutions on protein folding. Amino acid sequences were taken from the ancestral state reconstruction analysis, and folding was predicted using the I-TASSER server. The protein models were manipulated in Jmol. Structure of the ancestral (*A*) POU_S_ and (*B*) POU_HD_ domains. (*C–H*) Comparisons of protein folding in the ancestral sequences versus ancestral POU family members for significant amino acid substitutions to known DNA-binding sites. All measurements are in nanometers. (*C*) Ancestral condition of position 60. (*D*) Derived condition of position 60 in the last common ancestor of POU4. (*E*) Ancestral condition of position 64. (*F*) Derived condition of position 64 in the last common ancestor of *POU4*. (*G*) Ancestral condition of position 91. (*H*) Derived condition of position 91 in the last common ancestor of *POU6*.

Given the modular nature of POU genes, we were curious whether there was any evidence of some modules evolving at different rates than others. Mutations appear to be fairly evenly distributed between POU_S_ and POU_HD_ domains at every node, but more mutations occur in α-helices in the POU_HD_ domain (66 of 87 amino acid changes for POU_HD_ vs. 51 of 86 changes for POU_S_), even though the α-helix portion of POU_HD_ is smaller than in POU_S_. As mentioned earlier, most amino acids known to play a direct role in DNA binding are largely invariant across the gene family. However, there are several significant amino acid substitutions in *POU4* (60K→N 64K→A) and *POU6* (91K→L) at positions involved in DNA binding in other POU classes. The consequences of these substitutions are unclear; the crystal structure has not been studied in *POU4* or *POU6*, so the impact that these substitutions have on DNA binding/bending is unknown. The protein folding prediction software I-TASSER (Roy et al. 2010) suggests that these substitutions have a minor impact on the shape of α-helices (fig. 4).

**Fig. 5.**
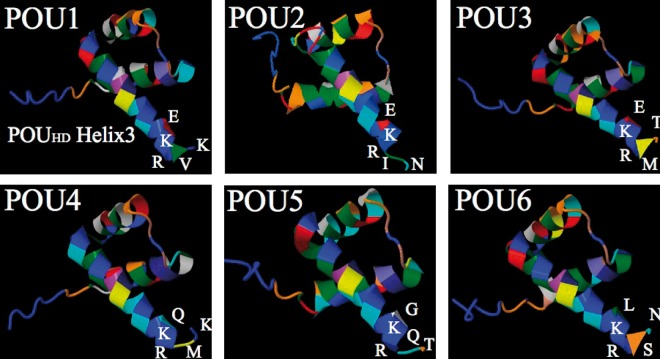
Predicted folding of POU_HD_ domains in the last common ancestor of each POU family, with a focus on the C-terminus. The last common ancestors of *POU1*, *POU3*, and *POU6* exhibit C-termini that are similar to the ancestral POU protein (see fig. 4*B*). In contrast, the last common ancestors of *POU2*, *POU4*, and *POU5* display an unwinding of the final dipeptide from the recognition α-helix.

The last two amino acids of the POU_HD_ domain are distinct at each family-level bifurcation, and in four of the six cases there is a conserved combination of an aliphatic residue followed by a charged residue. This supports the hypothesis that this dipeptide is important in driving functional differentiation between the classes (Jauch et al. 2010). In *POU1*, amino acids 135–138 sit in an extended conformation beyond the terminus of the alpha helix (Jacobson et al. 1997), which is likely critical in driving dimerization in the final dipeptide. Thus, there might be an implicit loss of dimerization specification in *POU6* and *POU5*, the two families that have lost this aliphatic/charged motif in the final dipeptide. Position 134, 2 bp upstream of this final dipeptide, also exhibits an interesting evolutionary pattern; at each bifurcation, the ancestral peptide (glutamic acid) is retained in one lineage, whereas the other lineage exhibits a significant substitution (*POU6* E→L, *POU4* E→Q, *POU5* E→G). Protein folding predictions of the POU_HD_ domain suggest that these substitutions have impacted the conformation of the recognition helix C-terminus (fig. 5); *POU6*, *POU1*, and *POU3* have retained the structure of the ancestral POU_HD_ protein (see fig. 4*B*), whereas *POU2*, *POU4*, and *POU5* exhibit an unwinding of the final dipeptide.

## Discussion

**Fig. 6.**
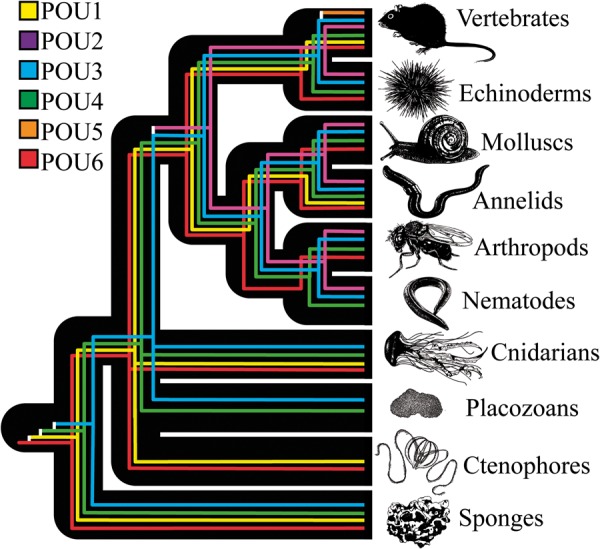
Reconciliation of the POU gene tree and our animal phylogeny. This figure summarizes our hypothesis regarding how major POU families were gained and lost across the major animal phyla. Presence/absence results as they retain to each phylum were verified using BLAST searches on NCBI and through publically available genome data sets. Some of the animal images in this figure were modified under the creative commons agreement from the OpenLearn Tree of Life project (http://www.open.edu/openlearn/nature-environment/natural-history/tree-life, last accessed August 24, 2014). The base of the animal tree, particularly the placement of ctenophores and placozoans, is an active area of research. Opposing animal phylogenies to the one we present here have the potential to alter how rapid this initial expansion of POU classes was, but all currently debated animal topologies would still require the divergence of the first four POU genes prior to the evolution of the *Eumetozoa*.

The results of this study are summarized in figure 6. The POU gene tree is marked by an early diversification, followed by significant gene loss in multiple clades. Our study suggests that two major events shaped the evolution of POU genes at the family-level: The radiation of POU paralogs prior to the evolution of the Bilateria and the evolution of *POU5* in the vertebrates.

If we accept sponges as the earliest-branching animal clade, then four of the six POU families had evolved before the last common ancestor of living animals (*POU6*, *POU1*, *POU3*, and *POU4*), with a fifth family (*POU2*) evolving in the stem lineage leading to modern bilaterian animals. However, several recent phylogenomic studies have suggested that ctenophores represent the oldest living animals (Ryan et al. 2013; Moroz et al. 2014). Our gene phylogeny is consistent with this evolutionary scenario, as ctenophores have *POU6* and *POU1* homologs—the two original POU families determined in our analyses. In either evolutionary scenario, the lineage leading up to the sponges exhibited a dramatic increase in POU paralogs, which is in marked contrast to their simple bodyplans. This discrepancy could be explained by a secondary reduction of the sponge bodyplan from a more complex ancestor, or by the hypothesis that various eumetazoan organs regulated by POU genes share a deep common ancestry among the small number of cell types that sponges possess (reviewed in Jacobs and Gates 2001; Jacobs et al. 2007, 2010). Supporting the later hypothesis, reverse transcription PCR in *Ephydatia* shows that multiple POU genes are expressed during the period of canal formation, suggesting that they might serve overlapping roles in choanocyte formation (Seimiya et al. 1997). Additional work on resolving the base of the animal tree, alongside an increased study of POU genes in early-branching animals, should help resolve these competing hypotheses (e.g., see Nakanishi et al. 2010; Hroudova et al. 2012 for examples of POU-class gene expression in complex cnidarians).

Our results also constrain the evolution of the *POU5* family to the vertebrates, which is particularly germane to understanding of the evolutionary role of *POU5f1/Oct-4* in maintaining stem cell pluripotency. Frankenberg and Renfree (2013) previously suggested that *POU5* is a gnathostome novelty, as the gene is missing from the lamprey, lancelet, and tunicate genomes. However, significant lineage-specific gene loss has occurred following a pregnathostome duplication event; the *POU5* paralog *Pou2* (not to be confused with the *POU2* family; see table 1) was lost in eutherian mammals and squamates, whereas the paralog *POU5f1/Oct-4* was lost in teleost fish, archosaurs, and anurans (Niwa et al. 2008; Frankenberg et al. 2010; Frankenberg and Renfree 2013). It is uncertain whether all *POU5*-family genes play a functional role in stem cell pluripotency, or whether that function is restricted to *POU5f1/Oct-4.* Morrison and Brickman (2006) found that zebrafish *Pou2* has little to no ability to support the self-renewal of mouse embryonic stem cells, but *Pou2* from other taxa—including axolotl, medaka (Tapia et al. 2012), and to a lesser extent chicken (Lavial et al. 2007)—can regulate pluripotency in mammalian stem cells. This suggests that all vertebrate *POU5* paralogs can play a role in regulating pluripotency (Onichtchouk 2012; Tapia et al. 2012). However, major differences in mammalian and nonmammalian stem cell dynamics exist (Fernandez-Tresguerres et al. 2010; Esch et al. 2013), and the extent that *Pou2* can co-opt the role of *POU5f1* remains unclear. For example, the *Pou2* homolog from the medaka fish can reprogram human fibroblasts into pluripotent stem cells (Tapia et al. 2012), but replacing the linker region of human *POU5f1* with the medaka linker abolishes the gene’s ability to induce pluripotency (Esch et al. 2013).

Assuming all vertebrate *POU5* genes play some collective role in cell pluripotency, is it possible that they inherited this function from an ancestral invertebrate POU paralog? POU genes are involved in stem cell dynamics of the planarian worm *Schmidtea mediterranea* (Onal et al. 2012) as well as the cnidarian *Hydractinia echinata* (Millane et al. 2011). Although these taxa were not included in our analyses, the results of our study would suggest that these invertebrate genes do not represent genuine *POU5* orthologs. Adding these proteins to our phylogenetic alignment suggests that candidate *Schmidtea* and *Hydractinia* genes represent *POU4* and *POU3* paralogs, respectively (supplementary fig. S9, Supplementary Material online). Additionally, these invertebrate POU sequences lack the α-helix domain that exists in the linker of amniote *POU5* peptides (supplementary fig. S10, Supplementary Material online), which is necessary for inducing pluripotency in mammalian cells according to Esch et al. (2013). This could be interpreted as further evidence for the independent evolution of invertebrate and mammalian stem cells (Gold and Jacobs 2013), although additional regulatory and epigenetic similarities between planarian and mammalian stem cells suggest that there might still be deep underlying conservation of the pluripotency network, even if disparate POU paralogs are ultimately utilized in different animal lineages (Onal et al. 2012). Such uncertainty only reinforces the point that we are just beginning to appreciate how dynamically evolving protein families become integrated into ancestral and novel genetic networks.

In an era of comparative and functional genomics, the elucidation of gene trees will prove just as important as the resolution of species trees. Our results suggest that POU genes have undergone a complex series of lineage-specific duplication and loss, which will only be fully clarified by using an extensive and diverse sampling of animals. Greater study of POU genes in animal clades such as sponges, cnidarians, ctenophores, and annelids should help elucidate the functional evolution of the POU class, and will be critical to determining cellular homologies between the invertebrates and vertebrates. This will likely prove important for establishing invertebrate model systems for a variety of developmental phenomena, including neurogenesis and stem cell dynamics.

## Materials and Methods

### Data Collection and Alignment

For our phylogenetic analysis, we searched for POU sequences from the publically available genomes of *A. queenslandica* (demosponge)*, O. carmella* (homoscleromorph sponge)*, Hydra magnipapillata* (cnidarian), *Nematostella vectensis* (cnidarian), *M. leidiy* (ctenophore), *T. adherans* (placozoan)*, Cap. teleta* (annelid)*, Lottia gigantea* (mollusc), *Cae. elegans* (nematode), *Tribolium castaneum* (arthropod), *Drosophila melanogaster* (arthropod), *Strongylocentrotus purpuratus* (echinoderm), *Xenopus tropicalis* (vertebrate), *Mus musculus* (vertebrate), and *Homo sapiens* (vertebrate). We also included sequences based on our unpublished transcriptomic data for *Aurelia* sp.1 (cnidarian). Databases were queried using the Human *Pit-1* POU_S_ domain: DSPEIRELEKFANEFKVRRIKLGYTQTNVGEALAAVHGSEFSQTTICRFENLQLSFKNACKLKAILSKWL. Sequences from *Hydra*, *Nematostella*, *Lottia*, *Caenorhabditis*, *Tribolium*, *Drosophila*, *Strongylocentrotus*, *Xenopus*, *Mus*, and *Homo* were collected from Metazome (http://www.metazome.net/, last accessed August 24, 2014) using BLASTP against the predicted proteomes. For *A. queenslandica*, we used TBLASTN against the Spongezome Metazome database (http://spongezome.metazome.net, last accessed August 24, 2014). Sequences from *Capitella* and *Trichoplax* were collected from the Joint Genome Institute using BLASTP. *Oscarella* sequences were obtained from the predicted protein models (OCAR G-PEP) available on the Compagen website (Hemmrich and Bosch 2008). *Mnemiopsis* proteins were recovered using BLASTP against the protein models (v2.2) available at the NIH *Mnemiopsis* Genome Project Portal (http://research.nhgri.nih.gov/mnemiopsis/blast/, last accessed August 24, 2014). The proteins we recovered for *Amphimedon* and *Mnemiopsis* are not identical to those that have been previously published (Larroux et al. 2008; Ryan et al. 2010); we interpreted this as resulting from improvements in the respective genome/proteome assemblies, and chose to work with the POU proteins we recovered. For the *Capitella POU1* gene, we recovered an alternative transcript using TBLASTN against the genome, which contained part of the POU_S_ domain missing from the predicted peptide; this longer sequence was used for subsequent analyses. Accession numbers for all genes are included in the alignment, available as supplementary file S1, Supplementary Material online.

Sequences were aligned using the MUSCLE algorithm (Edgar 2004) in Geneious (v.5.4.6., created by Biomatters and available from http://www.geneious.com/​, last accessed August 24, 2014). The alignment was edited by hand and restricted to the POU_S_ and POU_HD_ domains. Redundant sequences, unalignable sequences, and uninformative (unique) insertions were manually removed. The final alignment is available as supplementary file S1, Supplementary Material online.

### Phylogenic Analyses

We used ProtTest3 (Darriba et al. 2011) to determine the best-fitting model of amino acid evolution for our alignments. The program strongly preferred the LG model in conjunction with a gamma distribution and four substitution rate categories. We used PhyML (Guindon et al. 2010) to perform maximum-likelihood estimates; node values were determined using approximate likelihood ratio tests (aLRT) with Shimodaira-Hasegawa (SH)-like support. We used PhyloBayes 3.3 (Lartillot et al. 2009) for our Bayesian analyses. PhyloBayes was ran with the commands “pb -d {Alignment} -lg -nchain 2 100 0.3 100 {Output},” which means that the program ran two chains in parallel, checking every 100 cycles to see whether all discrepancies between the two chains were less than or equal to 0.3, and that all effective sizes were larger than 100. The runs were automatically stopped once these conditions were met.

### Gene Fishing

Animals were starved for at least 48 h prior to sampling. Genomic DNA was extracted using either a classic C-Tab protocol (Bebenek et al. 2004) or the DNeasy Kit (Qiagen). Degenerate PCR primers were designed to capture conserved regions of the POU_S_ and POU_HD_ domains (F1: CAA GCA GMG RMG VAT MAA RYT RGG; F2: CTB ACB YTB TCV CAY AAC AAC ATG; R1: CKY TTY TCN GGH GCV GCR ATR S; R2: RTT RCA RAA CCA SAC BCK MAC MAC). For each gene recovered, we used BLAST as well as phylogenetic analysis (supplementary fig. S8, Supplementary Material online) to assign a family identity to each gene. These family identities were supported with MUSCLE-based alignments of the linker regions, performed in Geneious (supplementary fig. S7, Supplementary Material online).

### Ancestral State Reconstruction

Accurate ancestral state reconstruction requires a gene tree that is consistent with the species tree, which is not generally the result of a standard ML or Bayesian analysis. To generate a gene tree informed by the species tree, we created an additional topology using TreeBeST (Vilella et al. 2008). Because of uncertainties in the topology at the base of the animal tree, we removed *Oscarella*, *Mnemiopsis*, and *Trichoplax* from our ancestral state reconstruction. We invoked the commands “treebest best -f {Input tree} -o {Output tree} {Alignment},” which resulted in a gene tree that was reconciled with the species tree, rooted by minimizing the number of duplications and losses, and bootstrapped 100 times.

The output of TreeBeST did a good job at creating a gene tree that was consistent with the species tree, with one exception. It produced a topology in the *POU6* family where all bilaterian invertebrate *POU6* genes were derived from one of the two vertebrate homologs (data not shown). This scenario would require a duplication of *POU6* at the base of the bilaterians, with the same paralog being lost in every invertebrate clade. A more likely scenario is that there was a single *POU6* gene in invertebrate bilaterians, and this gene duplicated in the vertebrates; a scenario that occurred in *POU2*, *POU3*, and *POU4* families. To modify the TreeBeST topology and get adjusted initial branch lengths, we ran the original POU alignment through BEUTi/BEAST (Drummond et al. 2012) for 500,000 generations, constraining every node as a prior to reflect the TreeBeST topology with our modification. For this analysis, we ultimately decided to exclude *Amphimedon POUB* and a *Nematostella POU3* paralog, as both sequences were highly derived, and we wished to avoid biasing our ancestral states with these sequences. However, it is worth noting that when *Amphimedon POUB* was included in the TreeBest analysis, it grouped with *POU4*. The final tree used for ancestral state reconstruction is available as supplementary file S2, Supplementary Material online.

The modified consensus tree and the relevant protein alignment were imported into the FastML server (Ashkenazy et al. 2012), using the LG substitution model, optimization of branch lengths, and gamma distribution options. The probabilities of the ancestral POU sequence were graphically exported using the WebLogo (Crooks et al. 2004) function in FastML and recolored in Adobe Illustrator to be consistent with MacClade-style amino acid coloration (as seen in fig. 1). The most probable ancestral state at each relevant node was exported from the FastML output, and amino acid substitutions were determined manually.

### Tests of Asymmetric Functional Divergence

We tested for functional divergence following gene duplication using the DIVERGE (v3.0) package (Gu et al. 2013). The tree used for ancestral state reconstruction (supplementary file S2, Supplementary Material online) and the relevant sequences were imported into DIVERGE to calculate the coefficient of functional divergence (or ⊖) for each pairwise comparison between POU families. We performed tests for type-I functional divergence (differences in amino acid variability between POU families) and type-II functional divergence (differences in significant amino acid substitutions between families, using the “significance” criteria described earlier). *Z* values were calculated by dividing ⊖ by the standard error, and *P* values were determined using a two-tailed *Z*-score test (normal distribution test). The results of all tests are available in supplementary figure S11, Supplementary Material online.

## Supplementary Material

Supplementary figures S1–S11, file S1, and file S2 are available at *Molecular Biology and Evolution* online (http://www.mbe.oxfordjournals.org/).

Supplementary Data
