# Supplementary material for: The Early Expansion and Evolutionary Dynamics of POU Class Genes
Source: Mol Biol Evol. 2014 Sep 25;31(12):3136–47. doi: 10.1093/molbev/msu243 (PMC4245813; doi:10.1093/molbev/msu243)
Supplement: Supplementary Data [file supp_31_12_3136__index.html]

The Early Expansion and Evolutionary Dynamics of POU Class Genes — The Early Expansion and Evolutionary Dynamics of POU Class Genes — Supplementary Data 

# The Early Expansion and Evolutionary Dynamics of POU Class Genes

## Supplementary Data

files

**Files in this Data Supplement:**

- Supplementary Data - pdf file
- Supplementary Data - pdf file
- Supplementary Data - txt file
- Supplementary Data - txt file
