# Supplementary material for: The Early Expansion and Evolutionary Dynamics of POU Class Genes
Source: Mol Biol Evol. 2014 Sep 25;31(12):3136–47. doi: 10.1093/molbev/msu243 (PMC4245813; doi:10.1093/molbev/msu243)
Supplement: Supplementary Data [file supp_msu243_Gold_et_al_Supplimental_Figures_S1-S5.pdf]

Figure S1-S6: Trees from phylogenetic analysis of POU class genes.
All maximum likelihood analyses were done in PhyML with an LG model of amino acid substitution, and a gamma distribution with four substitution rate categories. Bayesian analyses were performed in PhyloBayes 3.3, running two chains in parallel, until discrepancies between the chains were less than or equal to 0.3, and that the effective sizes were larger than 100.
(S1) Maximum likelihood analysis without outgroup sequences.
(S2) Bayesian analysis without outgroup sequences.
(S3) Maximum likelihood analysis with outgroup sequences.
(S4) Bayesian analysis with outgroup sequences.
(S5) Maximum likelihood analysis with *Amphimedon*, *Oscarella*, and *Mnemiopsis* sequences excluded.
(S6) Bayesian analysis with *Amphimedon*, *Oscarella*, and *Mnemiopsis* sequences excluded.

Figure S7: Alignment of POU linker regions for POU1, POU3, and POU4.
Sequences with an asterisk (*) represent genes recovered from our gene fishing analysis.

Figure S8: Phylogenetic analysis of POU class genes, including sequences recovered from our gene fishing study.
The tree was generated using maximum likelihood through PhyML with an LG model of amino acid substitution, and a gamma distribution with four substitution rate categories. The probability of nodes was determined using 200 bootstraps. Sequences from the gene fishing study are colored red.

Figure S9: Phylogenetic analysis of POU class genes, including stem cell-specific POU genes from *Schmidtea mediterranea* and *Hydractinia echinata*.
The tree was generated using maximum likelihood through PhyML. Probability of nodes was determined using 200 bootstraps. Sequences from *Schmidtea* and *Hydractinia* are colored red and noted with arrows.

Figure S10: Alignment of POU linker regions for POU5, POU3, and stem cell-specific POU genes from *Schmidtea mediterranea* and *Hydractinia echinata*.
Note that all invertebrate *POU3* sequences lack the conserved features of the amniote POU5 linker that are responsible for maintaining stem cell pluripotency, including the critical glutamate at position six. Alternative alignments of *Schmidtea* and *Hydractinia* linker regions are possible, and their alignment to the vertebrate *POU5* linker in this figure is not meant to suggest that they represent *POU5* homologs.

Figure S11: Results of type-I and type-II tests of asymmetric gene family evolution following gene duplication.
The values for the coefficient of functional divergence (ϴ) and the standard error for each pairwise comparison was performed in DIVERGE (v3.0).

Figure S1

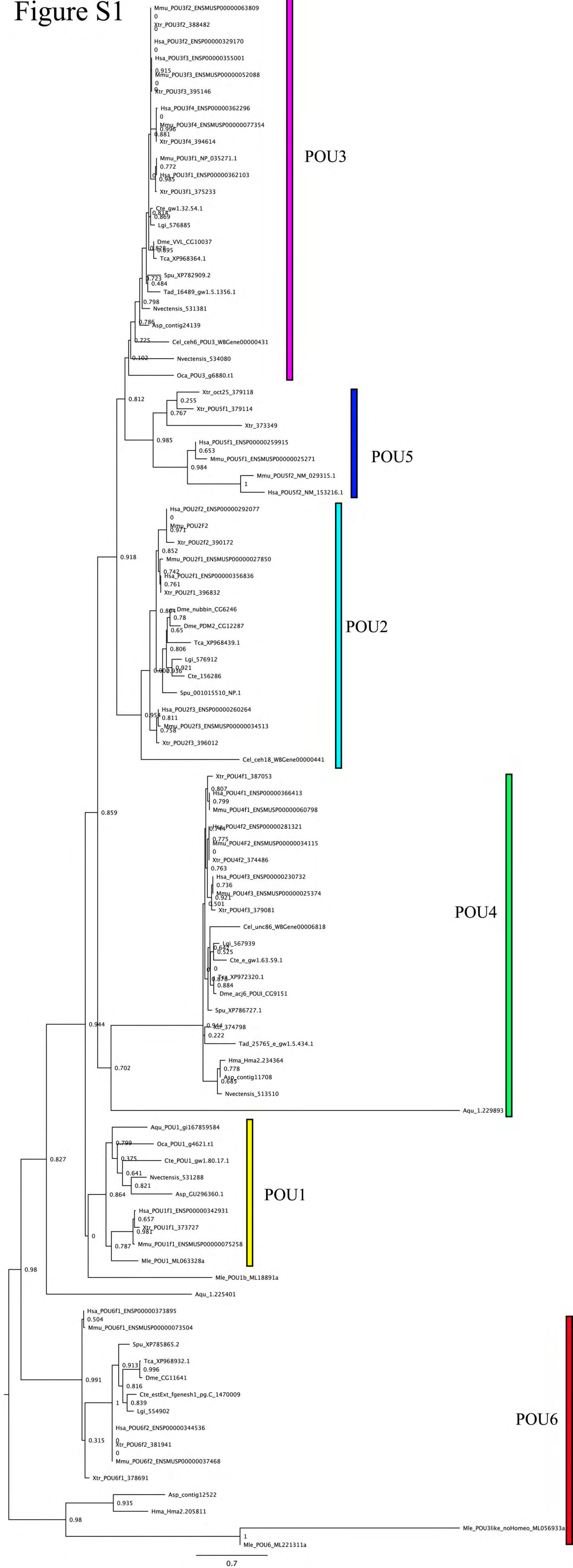
Mmu_POU3f2_ENSMUSP00000063809
Xtr_POU3f2_388482
Hsa_POU3f2_ENSP00000329170
Hsa_POU3f3_ENSP00000355001
Mmu_POU3f3_ENSMUSP00000052088
Xtr_POU3f3_395146
Hsa_POU3f4_ENSP00000362296
Mmu_POU3f4_ENSMUSP00000077354
Xtr_POU3f4_394614
Mmu_POU3f1_NP_035271.1
Hsa_POU3f1_ENSP00000362103
Xtr_POU3f1_375233
Cte_gw1.32.54.1
Lgi_576885
Dme_VVL_CG10037
Tca_XP968364.1
Spu_XP782909.2
Tad_16489_gw1.5.1356.1
Nvectensis_531381
Asp_contig24139
Cel_ceh6_POU3_WBGene00000431
Nvectensis_534080
Oca_POU3_g6880.t1
POU3
Xtr_oct25_379118
Xtr_POU5f1_379114
Xtr_373349
Hsa_POU5f1_ENSP00000259915
Mmu_POU5f1_ENSMUSP00000025271
Mmu_POU5f2_NM_029315.1
Hsa_POU5f2_NM_153216.1
POU5
Hsa_POU2f2_ENSP00000292077
Mmu_POU2F2
Xtr_POU2f2_390172
Mmu_POU2f1_ENSMUSP00000027850
Hsa_POU2f1_ENSP00000356836
Xtr_POU2f1_396832
Dme_nubbin_CG6246
Dme_PDM2_CG12287
Tca_XP968439.1
Lgi_576912
Cte_156286
Spu_001015510_NP.1
Hsa_POU2f3_ENSP00000260264
Mmu_POU2f3_ENSMUSP00000034513
Xtr_POU2f3_396012
Cel_ceh18_WBGene00000441
POU2
Xtr_POU4f1_387053
Hsa_POU4f1_ENSP00000366413
Mmu_POU4f1_ENSMUSP00000060798
Hsa_POU4f2_ENSP00000281321
Mmu_POU4F2_ENSMUSP00000034115
Xtr_POU4f2_374486
Hsa_POU4f3_ENSP00000230732
Mmu_POU4f3_ENSMUSP00000025374
Xtr_POU4f3_379081
Cel_unc86_WBGene00006818
Lgi_567939
Cte_e_gw1.63.59.1
Tca_XP972320.1
Dme_acj6_POUI_CG9151
Spu_XP786727.1
Xtr_374798
Tad_25765_e_gw1.5.434.1
Hma_Hma2.234364
Asp_contig11708
Nvectensis_513510
Aqu_1.229893
POU4
Aqu_POU1_gi167859584
Oca_POU1_g4621.t1
Cte_POU1_gw1.80.17.1
Nvectensis_531288
Asp_GU296360.1
Hsa_POU1f1_ENSP00000342931
Xtr_POU1f1_373727
Mmu_POU1f1_ENSMUSP00000075258
Mle_POU1_ML063328a
POU1
Mle_POU1b_ML18891a
Aqu_1.225401
Hsa_POU6f1_ENSP00000373895
Mmu_POU6f1_ENSMUSP00000073504
Spu_XP785865.2
Tca_XP968932.1
Dme_CG11641
Cte_estExt_fgenesh1_pg.C_1470009
Lgi_554902
Hsa_POU6f2_ENSP00000344536
Xtr_POU6f2_381941
Mmu_POU6f2_ENSMUSP00000037468
Xtr_POU6f1_378691
POU6
Asp_contig12522
Hma_Hma2.205811
Mle_POU3like_noHomeo_ML056933a
Mle_POU6_ML221311a
0.7

Figure S2

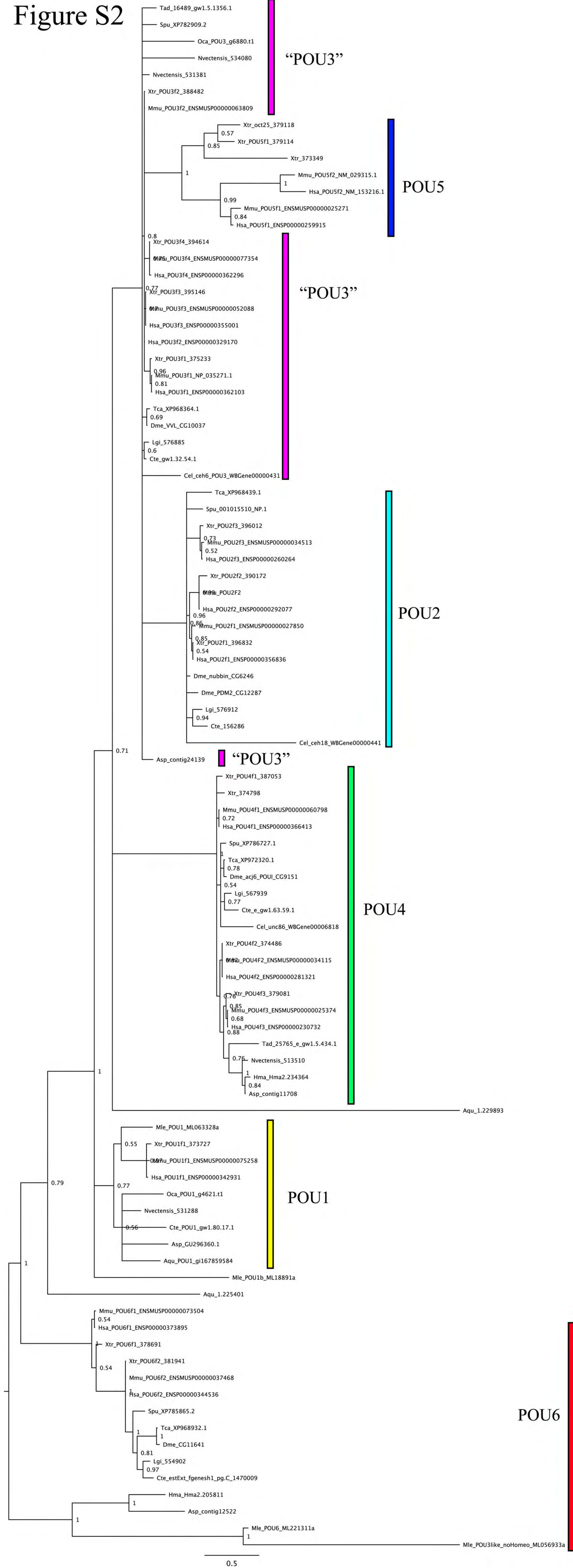

"POU3"
POU5
"POU3"
POU2
"POU3"
POU4
POU1
POU6
Tad_16489_gw1.5.1356.1
Spu_XP782909.2
Oca_POU3_g6880.t1
Nvectensis_534080
Nvectensis_531381
Xtr_POU3f2_388482
Mmu_POU3f2_ENSMUSP00000063809
Xtr_oct25_379118
Xtr_POU5f1_379114
Xtr_373349
Mmu_POU5f2_NM_029315.1
Hsa_POU5f2_NM_153216.1
Mmu_POU5f1_ENSMUSP00000025271
Hsa_POU5f1_ENSP00000259915
Xtr_POU3f4_394614
Mmu_POU3f4_ENSMUSP00000077354
Hsa_POU3f4_ENSP00000362296
Xtr_POU3f3_395146
Mmu_POU3f3_ENSMUSP00000052088
Hsa_POU3f3_ENSP00000355001
Hsa_POU3f2_ENSP00000329170
Xtr_POU3f1_375233
Mmu_POU3f1_NP_035271.1
Hsa_POU3f1_ENSP00000362103
Tca_XP968364.1
Dme_VVL_CG10037
Lgi_576885
Cte_gw1.32.54.1
Cel_ceh6_POU3_WBGene00000431
Tca_XP968439.1
Spu_001015510_NP.1
Xtr_POU2f3_396012
Mmu_POU2f3_ENSMUSP00000034513
Hsa_POU2f3_ENSP00000260264
Xtr_POU2f2_390172
Mmu_POU2F2
Hsa_POU2f2_ENSP00000292077
Mmu_POU2f1_ENSMUSP00000027850
Xtr_POU2f1_396832
Hsa_POU2f1_ENSP00000356836
Dme_nubbin_CG6246
Dme_PDM2_CG12287
Lgi_576912
Cte_156286
Cel_ceh18_WBGene00000441
Asp_contig24139
Xtr_POU4f1_387053
Xtr_374798
Mmu_POU4f1_ENSMUSP00000060798
Hsa_POU4f1_ENSP00000366413
Spu_XP786727.1
Tca_XP972320.1
Dme_acj6_POUI_CG9151
Lgi_567939
Cte_e_gw1.63.59.1
Cel_unc86_WBGene00006818
Xtr_POU4f2_374486
Mmu_POU4F2_ENSMUSP00000034115
Hsa_POU4f2_ENSP00000281321
Xtr_POU4f3_379081
Mmu_POU4f3_ENSMUSP00000025374
Hsa_POU4f3_ENSP00000230732
Tad_25765_e_gw1.5.434.1
Nvectensis_513510
Hma_Hma2.234364
Asp_contig11708
Aqu_1.229893
Mle_POU1_ML063328a
Xtr_POU1f1_373727
Mmu_POU1f1_ENSMUSP00000075258
Hsa_POU1f1_ENSP00000342931
Oca_POU1_g4621.t1
Nvectensis_531288
Cte_POU1_gw1.80.17.1
Asp_GU296360.1
Aqu_POU1_gi167859584
Mle_POU1b_ML18891a
Aqu_1.225401
Mmu_POU6f1_ENSMUSP00000073504
Hsa_POU6f1_ENSP00000373895
Xtr_POU6f1_378691
Xtr_POU6f2_381941
Mmu_POU6f2_ENSMUSP00000037468
Hsa_POU6f2_ENSP00000344536
Spu_XP785865.2
Tca_XP968932.1
Dme_CG11641
Lgi_554902
Cte_estExt_fgenesh1_pg.C_1470009
Hma_Hma2.205811
Asp_contig12522
Mle_POU6_ML221311a
Mle_POU3like_noHomeo_ML056933a
0.5

Figure S3

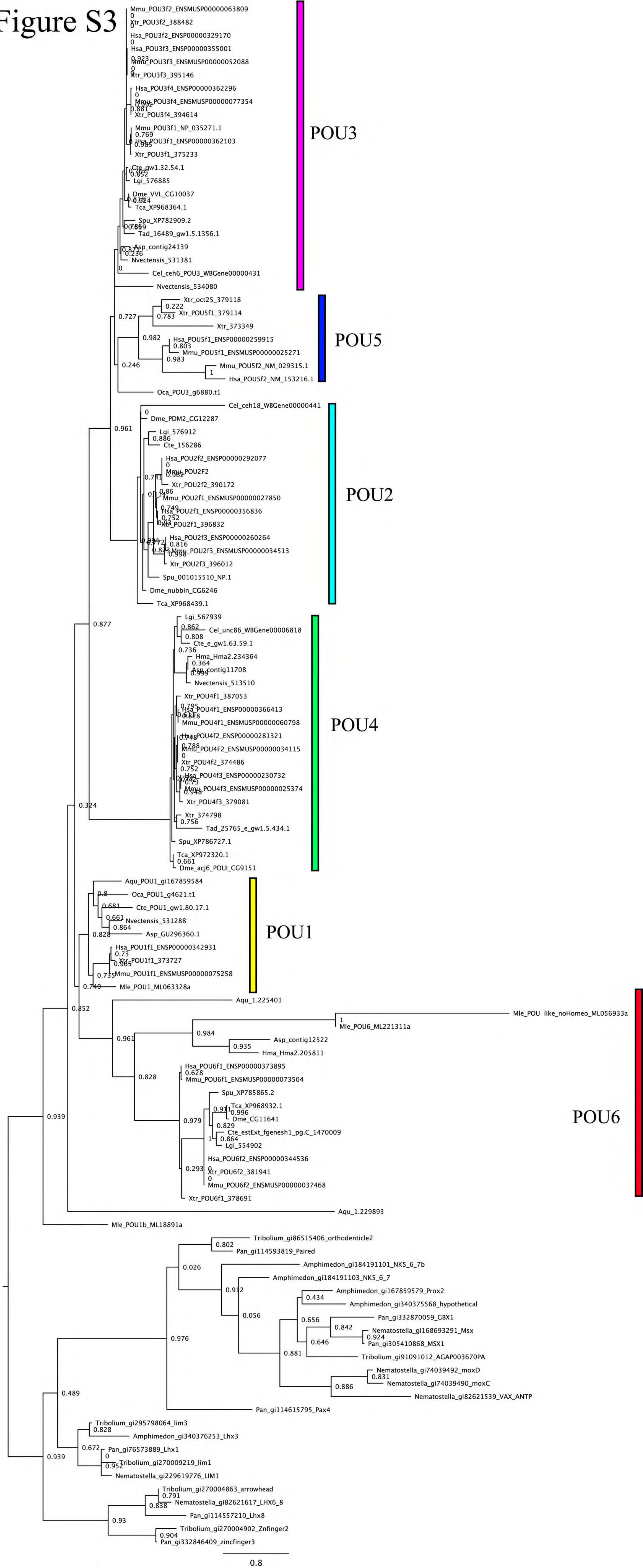
Mmu_POU3f2_ENSMUSP00000063809
Xtr_POU3f2_388482
Hsa_POU3f2_ENSP00000329170
Hsa_POU3f3_ENSP00000355001
Mmu_POU3f3_ENSMUSP00000052088
Xtr_POU3f3_395146
Hsa_POU3f4_ENSP00000362296
Mmu_POU3f4_ENSMUSP00000077354
Xtr_POU3f4_394614
Mmu_POU3f1_NP_035271.1
Hsa_POU3f1_ENSP00000362103
Xtr_POU3f1_375233
Cte_gw1.32.54.1
Lgi_576885
Dme_VVL_CG10037
Tca_XP968364.1
Spu_XP782909.2
Tad_16489_gw1.5.1356.1
Asp_contig24139
Nvectensis_531381
Cel_ceh6_POU3_WBGene00000431
Nvectensis_534080
POU3
Xtr_oct25_379118
Xtr_POU5f1_379114
Xtr_373349
Hsa_POU5f1_ENSP00000259915
Mmu_POU5f1_ENSMUSP00000025271
Mmu_POU5f2_NM_029315.1
Hsa_POU5f2_NM_153216.1
Oca_POU3_g6880.t1
POU5
Cel_ceh18_WBGene00000441
Dme_PDM2_CG12287
Lgi_576912
Cte_156286
Hsa_POU2f2_ENSP00000292077
Mmu_POU2F2
Xtr_POU2f2_390172
Mmu_POU2f1_ENSMUSP00000027850
Hsa_POU2f1_ENSP00000356836
Xtr_POU2f1_396832
Hsa_POU2f3_ENSP00000260264
Mmu_POU2f3_ENSMUSP00000034513
Xtr_POU2f3_396012
Spu_001015510_NP.1
Dme_nubbin_CG6246
Tca_XP968439.1
POU2
Lgi_567939
Cel_unc86_WBGene00006818
Cte_e_gw1.63.59.1
Hma_Hma2.234364
Asp_contig11708
Nvectensis_513510
Xtr_POU4f1_387053
Hsa_POU4f1_ENSP00000366413
Mmu_POU4f1_ENSMUSP00000060798
Hsa_POU4f2_ENSP00000281321
Mmu_POU4F2_ENSMUSP00000034115
Xtr_POU4f2_374486
Hsa_POU4f3_ENSP00000230732
Mmu_POU4f3_ENSMUSP00000025374
Xtr_POU4f3_379081
Xtr_374798
Tad_25765_e_gw1.5.434.1
Spu_XP786727.1
Tca_XP972320.1
Dme_acj6_POUI_CG9151
POU4
Aqu_POU1_gi167859584
Oca_POU1_g4621.t1
Cte_POU1_gw1.80.17.1
Nvectensis_531288
Asp_GU296360.1
Hsa_POU1f1_ENSP00000342931
Xtr_POU1f1_373727
Mmu_POU1f1_ENSMUSP00000075258
Mle_POU1_ML063328a
POU1
Aqu_1.225401
Mle_POU_like_noHomeo_ML056933a
Mle_POU6_ML221311a
Asp_contig12522
Hma_Hma2.205811
Hsa_POU6f1_ENSP00000373895
Mmu_POU6f1_ENSMUSP00000073504
Spu_XP785865.2
Tca_XP968932.1
Dme_CG11641
Cte_estExt_fgenesh1_pg.C_1470009
Lgi_554902
Hsa_POU6f2_ENSP00000344536
Xtr_POU6f2_381941
Mmu_POU6f2_ENSMUSP00000037468
Xtr_POU6f1_378691
POU6
Aqu_1.229893
Mle_POU1b_ML18891a
Tribolium_gi86515406_orthodenticle2
Pan_gi114593819_Paired
Amphimedon_gi184191101_NK5_6_7b
Amphimedon_gi184191103_NK5_6_7
Amphimedon_gi167859579_Prox2
Amphimedon_gi340375568_hypothetical
Pan_gi332870059_GBX1
Nematostella_gi168693291_Msx
Pan_gi305410868_MSX1
Tribolium_gi91091012_AGAP003670PA
Nematostella_gi74039492_moxD
Nematostella_gi74039490_moxC
Nematostella_gi82621539_VAX_ANTP
Pan_gi114615795_Pax4
Tribolium_gi295798064_lim3
Amphimedon_gi340376253_Lhx3
Pan_gi76573889_Lhx1
Tribolium_gi270009219_lim1
Nematostella_gi229619776_LIM1
Tribolium_gi270004863_arrowhead
Nematostella_gi82621617_LHX6_8
Pan_gi114557210_Lhx8
Tribolium_gi270004902_Znfinger2
Pan_gi332846409_zincfinger3
0.8

Figure S4

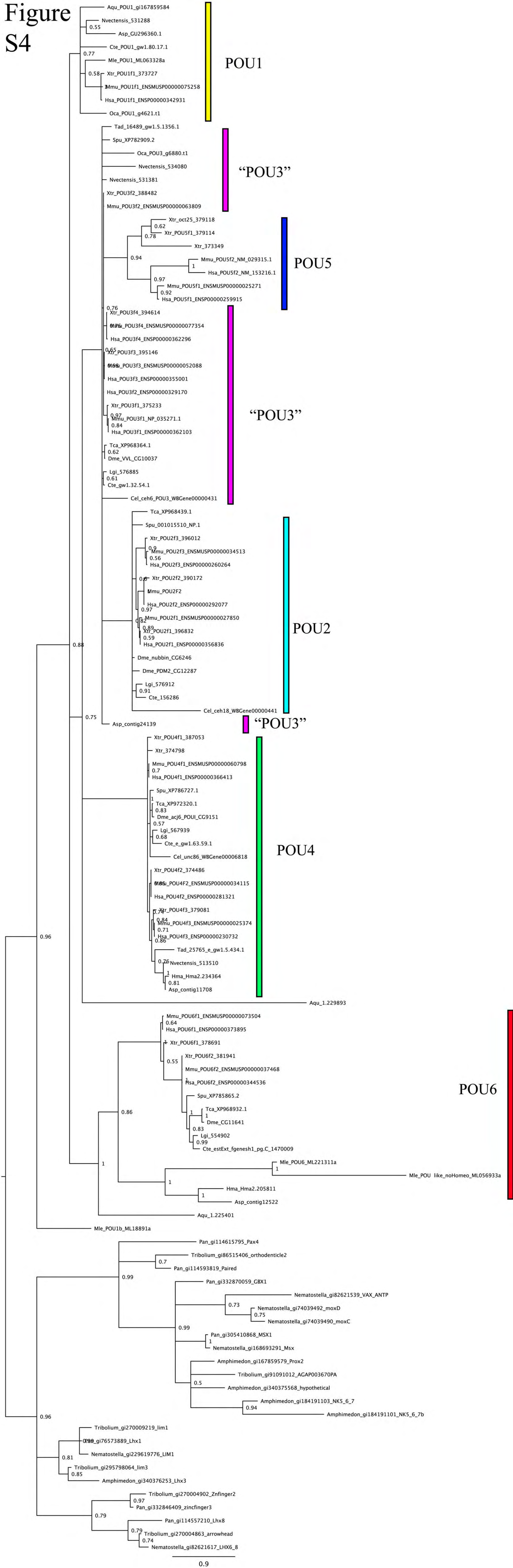
Aqu_POU1_gi167859584
0.55
Nvectensis_531288
Asp_GU296360.1
0.77
Cte_POU1_gw1.80.17.1
Mle_POU1_ML063328a
0.58
Xtr_POU1f1_373727
1
Mmu_POU1f1_ENSMUSP00000075258
Hsa_POU1f1_ENSP00000342931
Oca_POU1_g4621.t1
POU1
Tad_16489_gw1.5.1356.1
Spu_XP782909.2
Oca_POU3_g6880.t1
Nvectensis_534080
Nvectensis_531381
Xtr_POU3f2_388482
Mmu_POU3f2_ENSMUSP00000063809
"POU3"
Xtr_oct25_379118
0.62
0.78
Xtr_POU5f1_379114
Xtr_373349
0.94
Mmu_POU5f2_NM_029315.1
1
Hsa_POU5f2_NM_153216.1
0.97
Mmu_POU5f1_ENSMUSP00000025271
0.92
Hsa_POU5f1_ENSP00000259915
POU5
0.76
Xtr_POU3f4_394614
Mmu_POU3f4_ENSMUSP00000077354
Hsa_POU3f4_ENSP00000362296
0.65
Xtr_POU3f3_395146
Mmu_POU3f3_ENSMUSP00000052088
Hsa_POU3f3_ENSP00000355001
Hsa_POU3f2_ENSP00000329170
Xtr_POU3f1_375233
0.97
Mmu_POU3f1_NP_035271.1
0.84
Hsa_POU3f1_ENSP00000362103
Tca_XP968364.1
0.62
Dme_VVL_CG10037
Lgi_576885
0.61
Cte_gw1.32.54.1
Cel_ceh6_POU3_WBGene00000431
"POU3"
Tca_XP968439.1
Spu_001015510_NP.1
Xtr_POU2f3_396012
0.9
Mmu_POU2f3_ENSMUSP00000034513
0.56
Hsa_POU2f3_ENSP00000260264
Xtr_POU2f2_390172
Mmu_POU2F2
Hsa_POU2f2_ENSP00000292077
0.97
Mmu_POU2f1_ENSMUSP00000027850
0.89
Xtr_POU2f1_396832
0.59
Hsa_POU2f1_ENSP00000356836
Dme_nubbin_CG6246
Dme_PDM2_CG12287
Lgi_576912
0.91
Cte_156286
Cel_ceh18_WBGene00000441
POU2
0.88
0.75
Asp_contig24139
"POU3"
Xtr_POU4f1_387053
Xtr_374798
Mmu_POU4f1_ENSMUSP00000060798
0.7
Hsa_POU4f1_ENSP00000366413
Spu_XP786727.1
1
Tca_XP972320.1
0.83
Dme_acj6_POUI_CG9151
0.57
Lgi_567939
0.68
Cte_e_gw1.63.59.1
Cel_unc86_WBGene00006818
Xtr_POU4f2_374486
Mmu_POU4F2_ENSMUSP00000034115
Hsa_POU4f2_ENSP00000281321
Xtr_POU4f3_379081
0.84
Mmu_POU4f3_ENSMUSP00000025374
0.71
Hsa_POU4f3_ENSP00000230732
0.86
Tad_25765_e_gw1.5.434.1
0.76
Nvectensis_513510
1
Hma_Hma2.234364
0.81
Asp_contig11708
POU4
0.96
Aqu_1.229893
Mmu_POU6f1_ENSMUSP00000073504
0.64
Hsa_POU6f1_ENSP00000373895
1
Xtr_POU6f1_378691
0.55
Xtr_POU6f2_381941
Mmu_POU6f2_ENSMUSP00000037468
1
Hsa_POU6f2_ENSP00000344536
Spu_XP785865.2
1
Tca_XP968932.1
1
Dme_CG11641
0.83
Lgi_554902
0.99
Cte_estExt_fgenesh1_pg.C_1470009
0.86
Mle_POU6_ML221311a
1
Mle_POU like_noHomeo_ML056933a
1
1
Hma_Hma2.205811
1
Asp_contig12522
Aqu_1.225401
POU6
Mle_POU1b_ML18891a
Pan_gi114615795_Pax4
Tribolium_gi86515406_orthodenticle2
0.7
Pan_gi114593819_Paired
0.99
Pan_gi332870059_GBX1
Nematostella_gi82621539_VAX_ANTP
0.73
Nematostella_gi74039492_moxD
0.75
Nematostella_gi74039490_moxC
0.99
Pan_gi305410868_MSX1
1
Nematostella_gi168693291_Msx
Amphimedon_gi167859579_Prox2
Tribolium_gi91091012_AGAP003670PA
0.5
Amphimedon_gi340375568_hypothetical
Amphimedon_gi184191103_NK5_6_7
0.94
Amphimedon_gi184191101_NK5_6_7b
0.96
Tribolium_gi270009219_lim1
Pan_gi76573889_Lhx1
0.81
Nematostella_gi229619776_LIM1
Tribolium_gi295798064_lim3
0.85
Amphimedon_gi340376253_Lhx3
Tribolium_gi270004902_Znfinger2
0.97
Pan_gi332846409_zincfinger3
0.79
Pan_gi114557210_Lhx8
0.79
Tribolium_gi270004863_arrowhead
0.74
Nematostella_gi82621617_LHX6_8
0.9

Figure S5

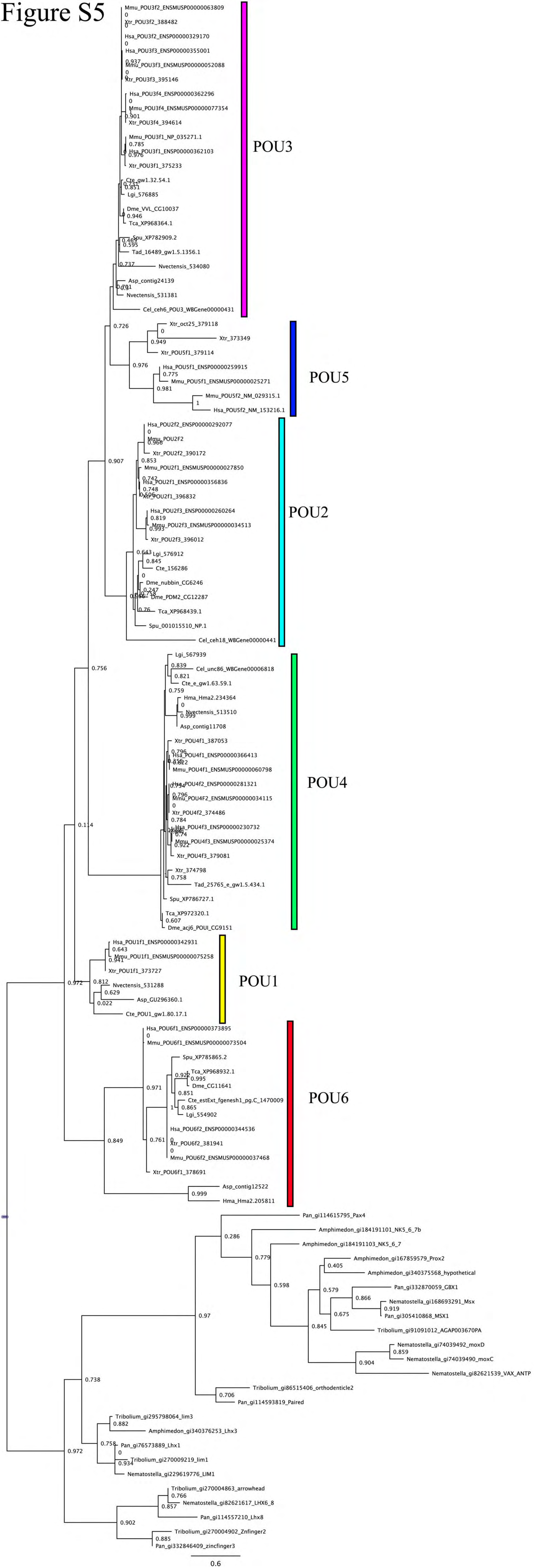

Mmu_POU3f2_ENSMUSP00000063809
Xtr_POU3f2_388482
Hsa_POU3f2_ENSP00000329170
Hsa_POU3f3_ENSP00000355001
Mmu_POU3f3_ENSMUSP00000052088
Xtr_POU3f3_395146
Hsa_POU3f4_ENSP00000362296
Mmu_POU3f4_ENSMUSP00000077354
Xtr_POU3f4_394614
Mmu_POU3f1_NP_035271.1
Hsa_POU3f1_ENSP00000362103
Xtr_POU3f1_375233
Cte_gw1.32.54.1
Lgi_576885
Dme_VVL_CG10037
Tca_XP968364.1
Spu_XP782909.2
Tad_16489_gw1.5.1356.1
Nvectensis_534080
Asp_contig24139
Nvectensis_531381
Cel_ceh6_POU3_WBGene00000431
POU3
Xtr_oct25_379118
Xtr_373349
Xtr_POU5f1_379114
Hsa_POU5f1_ENSP00000259915
Mmu_POU5f1_ENSMUSP00000025271
Mmu_POU5f2_NM_029315.1
Hsa_POU5f2_NM_153216.1
POU5
Hsa_POU2f2_ENSP00000292077
Mmu_POU2F2
Xtr_POU2f2_390172
Mmu_POU2f1_ENSMUSP00000027850
Hsa_POU2f1_ENSP00000356836
Xtr_POU2f1_396832
Hsa_POU2f3_ENSP00000260264
Mmu_POU2f3_ENSMUSP00000034513
Xtr_POU2f3_396012
Lgi_576912
Cte_156286
Dme_nubbin_CG6246
Dme_PDM2_CG12287
Tca_XP968439.1
Spu_001015510_NP.1
Cel_ceh18_WBGene00000441
POU2
Lgi_567939
Cel_unc86_WBGene00006818
Cte_e_gw1.63.59.1
Hma_Hma2.234364
Nvectensis_513510
Asp_contig11708
Xtr_POU4f1_387053
Hsa_POU4f1_ENSP00000366413
Mmu_POU4f1_ENSMUSP00000060798
Hsa_POU4f2_ENSP00000281321
Mmu_POU4F2_ENSMUSP00000034115
Xtr_POU4f2_374486
Hsa_POU4f3_ENSP00000230732
Mmu_POU4f3_ENSMUSP00000025374
Xtr_POU4f3_379081
Xtr_374798
Tad_25765_e_gw1.5.434.1
Spu_XP786727.1
Tca_XP972320.1
Dme_acj6_POUI_CG9151
POU4
Hsa_POU1f1_ENSP00000342931
Mmu_POU1f1_ENSMUSP00000075258
Xtr_POU1f1_373727
Nvectensis_531288
Asp_GU296360.1
Cte_POU1_gw1.80.17.1
POU1
Hsa_POU6f1_ENSP00000373895
Mmu_POU6f1_ENSMUSP00000073504
Spu_XP785865.2
Tca_XP968932.1
Dme_CG11641
Cte_estExt_fgenesh1_pg.C_1470009
Lgi_554902
Hsa_POU6f2_ENSP00000344536
Xtr_POU6f2_381941
Mmu_POU6f2_ENSMUSP00000037468
Xtr_POU6f1_378691
Asp_contig12522
Hma_Hma2.205811
POU6
Pan_gi114615795_Pax4
Amphimedon_gi184191101_NK5_6_7b
Amphimedon_gi184191103_NK5_6_7
Amphimedon_gi167859579_Prox2
Amphimedon_gi340375568_hypothetical
Pan_gi332870059_GBX1
Nematostella_gi168693291_Msx
Pan_gi305410868_MSX1
Tribolium_gi91091012_AGAP003670PA
Nematostella_gi74039492_moxD
Nematostella_gi74039490_moxC
Nematostella_gi82621539_VAX_ANTP
Tribolium_gi86515406_orthodenticle2
Pan_gi114593819_Paired
Tribolium_gi295798064_lim3
Amphimedon_gi340376253_Lhx3
Pan_gi76573889_Lhx1
Tribolium_gi270009219_lim1
Nematostella_gi229619776_LIM1
Tribolium_gi270004863_arrowhead
Nematostella_gi82621617_LHX6_8
Pan_gi114557210_Lhx8
Tribolium_gi270004902_Znfinger2
Pan_gi332846409_zincfinger3
0.6
