# Supplementary material for: The Early Expansion and Evolutionary Dynamics of POU Class Genes
Source: Mol Biol Evol. 2014 Sep 25;31(12):3136–47. doi: 10.1093/molbev/msu243 (PMC4245813; doi:10.1093/molbev/msu243)
Supplement: Supplementary Data [file supp_msu243_Gold_et_al_Supplimental_Figures_S6-S11.pdf]

Figure S1-S6: Trees from phylogenetic analysis of POU class genes.
All maximum likelihood analyses were done in PhyML with an LG model of amino acid substitution, and a gamma distribution with four substitution rate categories. Bayesian analyses were performed in PhyloBayes 3.3, running two chains in parallel, until discrepancies between the chains were less than or equal to 0.3, and that the effective sizes were larger than 100.
(S1) Maximum likelihood analysis without outgroup sequences.
(S2) Bayesian analysis without outgroup sequences.
(S3) Maximum likelihood analysis with outgroup sequences.
(S4) Bayesian analysis with outgroup sequences.
(S5) Maximum likelihood analysis with *Amphimedon*, *Oscarella*, and *Mnemiopsis* sequences excluded.
(S6) Bayesian analysis with *Amphimedon*, *Oscarella*, and *Mnemiopsis* sequences excluded.

Figure S7: Alignment of POU linker regions for POU1, POU3, and POU4.
Sequences with an asterisk (*) represent genes recovered from our gene fishing analysis.

Figure S8: Phylogenetic analysis of POU class genes, including sequences recovered from our gene fishing study.
The tree was generated using maximum likelihood through PhyML with an LG model of amino acid substitution, and a gamma distribution with four substitution rate categories. The probability of nodes was determined using 200 bootstraps. Sequences from the gene fishing study are colored red.

Figure S9: Phylogenetic analysis of POU class genes, including stem cell-specific POU genes from *Schmidtea mediterranea* and *Hydractinia echinata*.
The tree was generated using maximum likelihood through PhyML. Probability of nodes was determined using 200 bootstraps. Sequences from *Schmidtea* and *Hydractinia* are colored red and noted with arrows.

Figure S10: Alignment of POU linker regions for POU5, POU3, and stem cell-specific POU genes from *Schmidtea mediterranea* and *Hydractinia echinata*.
Note that all invertebrate *POU3* sequences lack the conserved features of the amniote POU5 linker that are responsible for maintaining stem cell pluripotency, including the critical glutamate at position six. Alternative alignments of *Schmidtea* and *Hydractinia* linker regions are possible, and their alignment to the vertebrate *POU5* linker in this figure is not meant to suggest that they represent *POU5* homologs.

Figure S11: Results of type-I and type-II tests of asymmetric gene family evolution following gene duplication.
The values for the coefficient of functional divergence (ϴ) and the standard error for each pairwise comparison was performed in DIVERGE (v3.0).

Figure S6

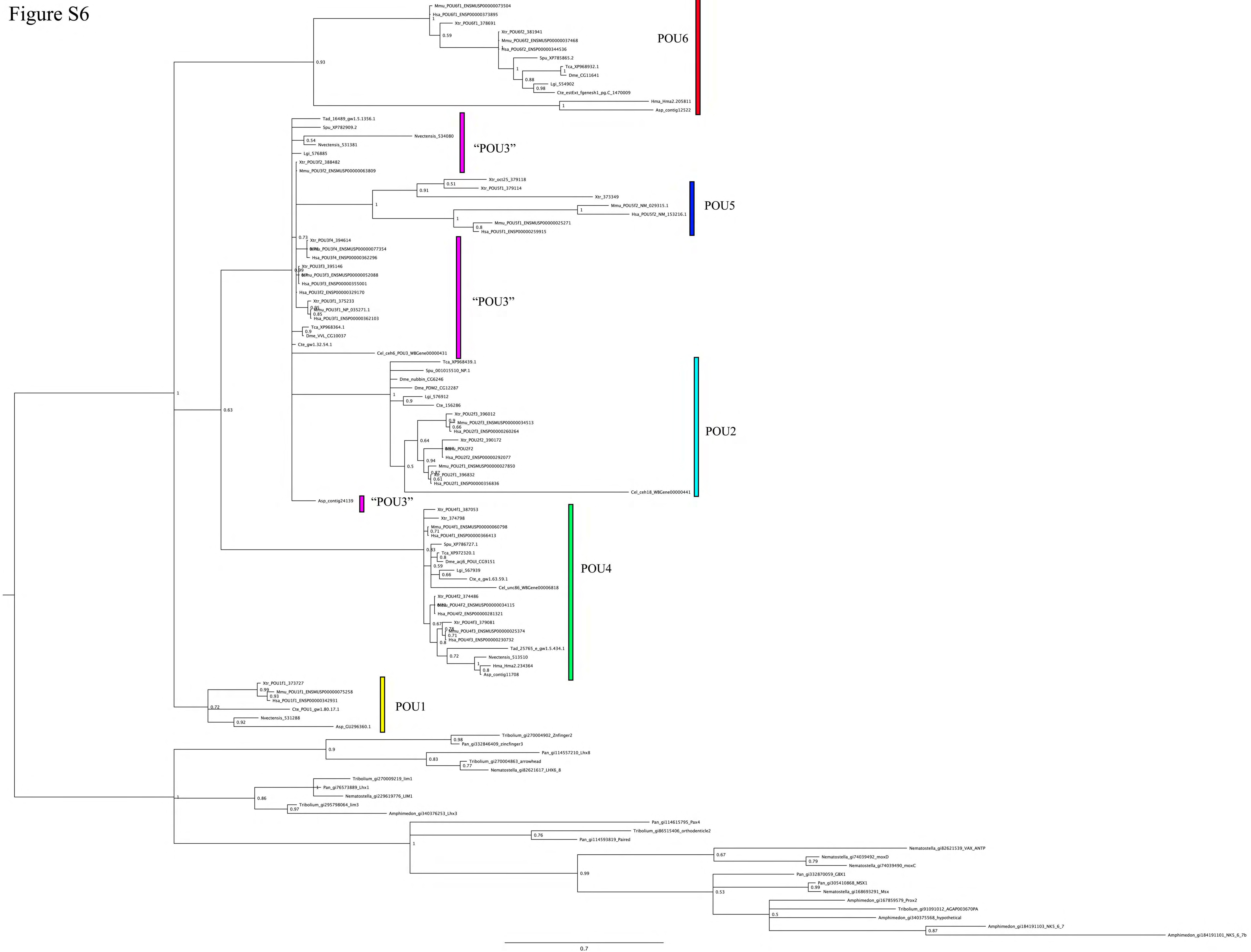
POU6
"POU3"
POU5
"POU3"
POU2
"POU3"
POU4
POU1
Mmu_POU6f1_ENSMUSP00000073504
Hsa_POU6f1_ENSP00000373895
Xtr_POU6f1_378691
Xtr_POU6f2_381941
Mmu_POU6f2_ENSMUSP00000037468
Hsa_POU6f2_ENSP00000344536
Spu_XP785865.2
Tca_XP968932.1
Dme_CG11641
Lgi_554902
Cte_estExt_fgenesh1_pg.C_1470009
Hma_Hma2.205811
Asp_contig12522
Tad_16489_gw1.5.1356.1
Spu_XP782909.2
Nvectensis_534080
Nvectensis_531381
Lgi_576885
Xtr_POU3f2_388482
Mmu_POU3f2_ENSMUSP00000063809
Xtr_oct25_379118
Xtr_POU5f1_379114
Xtr_373349
Mmu_POU5f2_NM_029315.1
Hsa_POU5f2_NM_153216.1
Mmu_POU5f1_ENSMUSP00000025271
Hsa_POU5f1_ENSP00000259915
Xtr_POU3f4_394614
Mmu_POU3f4_ENSMUSP00000077354
Hsa_POU3f4_ENSP00000362296
Xtr_POU3f3_395146
Mmu_POU3f3_ENSMUSP00000052088
Hsa_POU3f3_ENSP00000355001
Hsa_POU3f2_ENSP00000329170
Xtr_POU3f1_375233
Mmu_POU3f1_NP_035271.1
Hsa_POU3f1_ENSP00000362103
Tca_XP968364.1
Dme_VVL_CG10037
Cte_gw1.32.54.1
Cel_ceh6_POU3_WBGene00000431
Tca_XP968439.1
Spu_001015510_NP.1
Dme_nubbin_CG6246
Dme_PDM2_CG12287
Lgi_576912
Cte_156286
Xtr_POU2f3_396012
Mmu_POU2f3_ENSMUSP00000034513
Hsa_POU2f3_ENSP00000260264
Xtr_POU2f2_390172
Mmu_POU2F2
Hsa_POU2f2_ENSP00000292077
Mmu_POU2f1_ENSMUSP00000027850
Xtr_POU2f1_396832
Hsa_POU2f1_ENSP00000356836
Cel_ceh18_WBGene00000441
Asp_contig24139
Xtr_POU4f1_387053
Xtr_374798
Mmu_POU4f1_ENSMUSP00000060798
Hsa_POU4f1_ENSP00000366413
Spu_XP786727.1
Tca_XP972320.1
Dme_acj6_POUI_CG9151
Lgi_567939
Cte_e_gw1.63.59.1
Cel_unc86_WBGene00006818
Xtr_POU4f2_374486
Mmu_POU4F2_ENSMUSP00000034115
Hsa_POU4f2_ENSP00000281321
Xtr_POU4f3_379081
Mmu_POU4f3_ENSMUSP00000025374
Hsa_POU4f3_ENSP00000230732
Tad_25765_e_gw1.5.434.1
Nvectensis_513510
Hma_Hma2.234364
Asp_contig11708
Xtr_POU1f1_373727
Mmu_POU1f1_ENSMUSP00000075258
Hsa_POU1f1_ENSP00000342931
Cte_POU1_gw1.80.17.1
Nvectensis_531288
Asp_GU296360.1
Tribolium_gi270004902_Znfinger2
Pan_gi332846409_zincfinger3
Pan_gi114557210_Lhx8
Tribolium_gi270004863_arrowhead
Nematostella_gi82621617_LHX6_8
Tribolium_gi270009219_lim1
Pan_gi76573889_Lhx1
Nematostella_gi229619776_LIM1
Tribolium_gi295798064_lim3
Amphimedon_gi340376253_Lhx3
Pan_gi114615795_Pax4
Tribolium_gi86515406_orthodenticle2
Pan_gi114593819_Paired
Nematostella_gi82621539_VAX_ANTP
Nematostella_gi74039492_moxD
Nematostella_gi74039490_moxC
Pan_gi332870059_GBX1
Pan_gi305410868_MSX1
Nematostella_gi168693291_Msx
Amphimedon_gi167859579_Prox2
Tribolium_gi91091012_AGAP003670PA
Amphimedon_gi340375568_hypothetical
Amphimedon_gi184191103_NK5_6_7
Amphimedon_gi184191101_NK5_6_7b
0.7

Figure S7

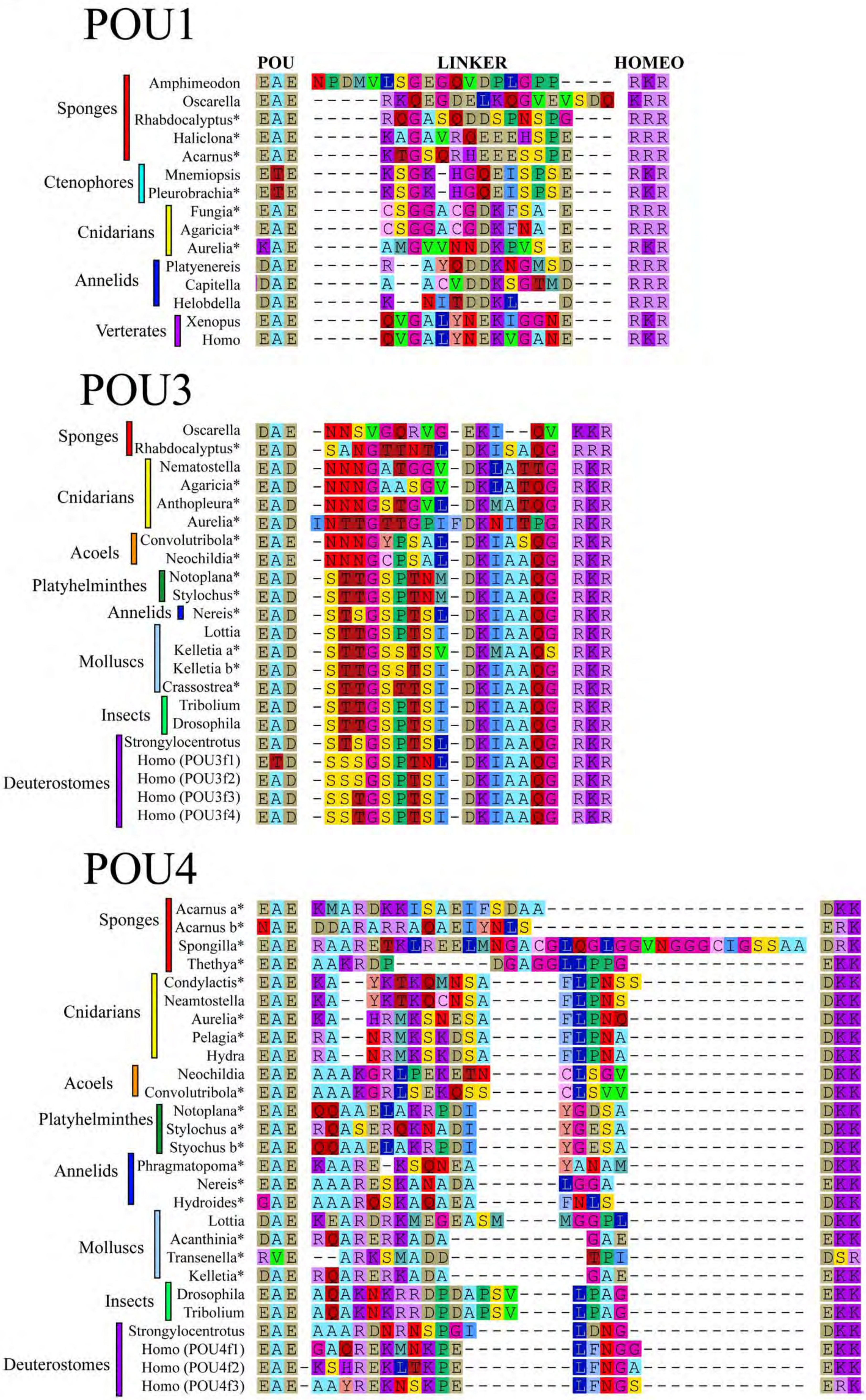

POU1
POU
LINKER
HOMEO
Sponges
Amphimeodon
Oscarella
Rhabdocalyptus*
Haliclona*
Acarnus*
Ctenophores
Mnemiopsis
Pleurobrachia*
Cnidarians
Fungia*
Agaricia*
Aurelia*
Annelids
Platyenereis
Capitella
Helobdella
Verterates
Xenopus
Homo
POU3
Sponges
Oscarella
Rhabdocalyptus*
Cnidarians
Nematostella
Agaricia*
Anthopleura*
Aurelia*
Acoels
Convolutribola*
Neochildia*
Platyhelminthes
Notoplana*
Stylochus*
Annelids
Nereis*
Molluscs
Lottia
Kelletia a*
Kelletia b*
Crassostrea*
Insects
Tribolium
Drosophila
Deuterostomes
Strongylocentrotus
Homo (POU3f1)
Homo (POU3f2)
Homo (POU3f3)
Homo (POU3f4)
POU4
Sponges
Acarnus a*
Acarnus b*
Spongilla*
Thethya*
Cnidarians
Condylactis*
Neamtostella
Aurelia*
Pelagia*
Hydra
Acoels
Neochildia
Convolutribola*
Platyhelminthes
Notoplana*
Stylochus a*
Styochus b*
Annelids
Phragmatopoma*
Nereis*
Hydroides*
Molluscs
Lottia
Acanthinia*
Transenella*
Kelletia*
Insects
Drosophila
Tribolium
Deuterostomes
Strongylocentrotus
Homo (POU4f1)
Homo (POU4f2)
Homo (POU4f3)

Figure S8

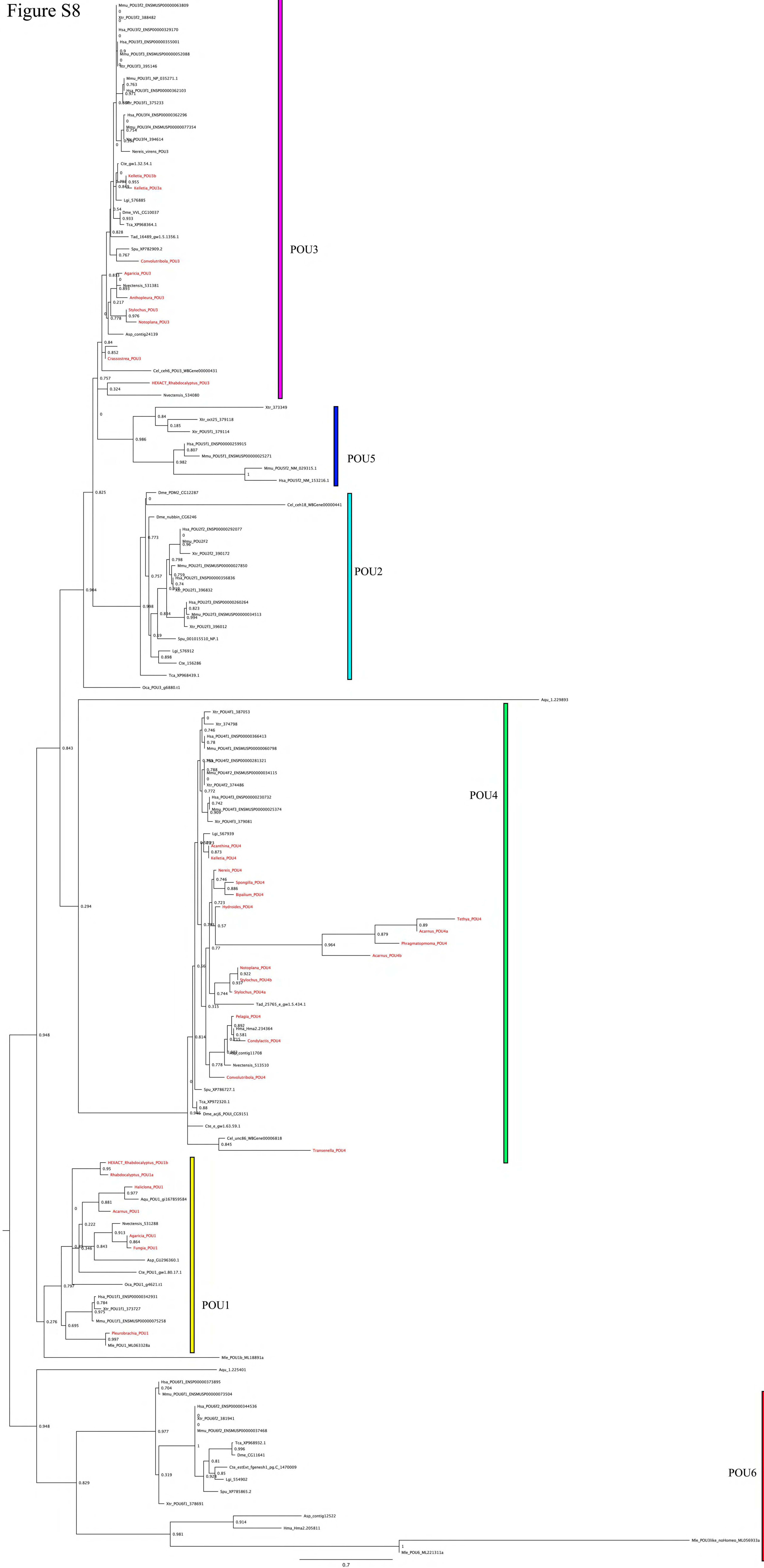

Mmu_POU3f2_ENSMUSP00000063809
Xtr_POU3f2_388482
Hsa_POU3f2_ENSP00000329170
Hsa_POU3f3_ENSP00000355001
Mmu_POU3f3_ENSMUSP00000052088
Xtr_POU3f3_395146
Mmu_POU3f1_NP_035271.1
Hsa_POU3f1_ENSP00000362103
Xtr_POU3f1_375233
Hsa_POU3f4_ENSP00000362296
Mmu_POU3f4_ENSMUSP00000077354
Xtr_POU3f4_394614
Nereis_virens_POU3
Cte_gw1.32.54.1
Kelletia_POU3b
Kelletia_POU3a
Lgi_576885
Dme_VVL_CG10037
Tca_XP968364.1
Tad_16489_gw1.5.1356.1
Spu_XP782909.2
Convolutribola_POU3
Agaricia_POU3
Nvectensis_531381
Anthopleura_POU3
Stylochus_POU3
Notoplana_POU3
Asp_contig24139
Crassostrea_POU3
Cel_ceh6_POU3_WBGene00000431
HEXACT_Rhabdocalyptus_POU3
Nvectensis_534080
POU3
Xtr_373349
Xtr_oct25_379118
Xtr_POU5f1_379114
Hsa_POU5f1_ENSP00000259915
Mmu_POU5f1_ENSMUSP00000025271
Mmu_POU5f2_NM_029315.1
Hsa_POU5f2_NM_153216.1
POU5
Dme_PDM2_CG12287
Cel_ceh18_WBGene00000441
Dme_nubbin_CG6246
Hsa_POU2f2_ENSP00000292077
Mmu_POU2F2
Xtr_POU2f2_390172
Mmu_POU2f1_ENSMUSP00000027850
Hsa_POU2f1_ENSP00000356836
Xtr_POU2f1_396832
Hsa_POU2f3_ENSP00000260264
Mmu_POU2f3_ENSMUSP00000034513
Xtr_POU2f3_396012
Spu_001015510_NP.1
Lgi_576912
Cte_156286
Tca_XP968439.1
Oca_POU3_g6880.t1
POU2
Aqu_1.229893
Xtr_POU4f1_387053
Xtr_374798
Hsa_POU4f1_ENSP00000366413
Mmu_POU4f1_ENSMUSP00000060798
Hsa_POU4f2_ENSP00000281321
Mmu_POU4F2_ENSMUSP00000034115
Xtr_POU4f2_374486
Hsa_POU4f3_ENSP00000230732
Mmu_POU4f3_ENSMUSP00000025374
Xtr_POU4f3_379081
Lgi_567939
Acanthina_POU4
Kelletia_POU4
Nereis_POU4
Spongilla_POU4
Bipalium_POU4
Hydroides_POU4
Tethya_POU4
Acarnus_POU4a
Phragmatopmoma_POU4
Acarnus_POU4b
Notoplana_POU4
Stylochus_POU4b
Stylochus_POU4a
Tad_25765_e_gw1.5.434.1
Pelagia_POU4
Hma_Hma2.234364
Condylactis_POU4
Asp_contig11708
Nvectensis_513510
Convolutribola_POU4
Spu_XP786727.1
Tca_XP972320.1
Dme_acj6_POUI_CG9151
Cte_e_gw1.63.59.1
Cel_unc86_WBGene00006818
Transenella_POU4
POU4
HEXACT_Rhabdocalyptus_POU1b
Rhabdocalyptus_POU1a
Haliclona_POU1
Aqu_POU1_gi167859584
Acarnus_POU1
Nvectensis_531288
Agaricia_POU1
Fungia_POU1
Asp_GU296360.1
Cte_POU1_gw1.80.17.1
Oca_POU1_g4621.t1
Hsa_POU1f1_ENSP00000342931
Xtr_POU1f1_373727
Mmu_POU1f1_ENSMUSP00000075258
Pleurobrachia_POU1
Mle_POU1_ML063328a
POU1
Mle_POU1b_ML18891a
Aqu_1.225401
Hsa_POU6f1_ENSP00000373895
Mmu_POU6f1_ENSMUSP00000073504
Hsa_POU6f2_ENSP00000344536
Xtr_POU6f2_381941
Mmu_POU6f2_ENSMUSP00000037468
Tca_XP968932.1
Dme_CG11641
Cte_estExt_fgenesh1_pg.C_1470009
Lgi_554902
Spu_XP785865.2
Xtr_POU6f1_378691
Asp_contig12522
Hma_Hma2.205811
Mle_POU3like_noHomeo_ML056933a
Mle_POU6_ML221311a
POU6
0.7

Figure S9

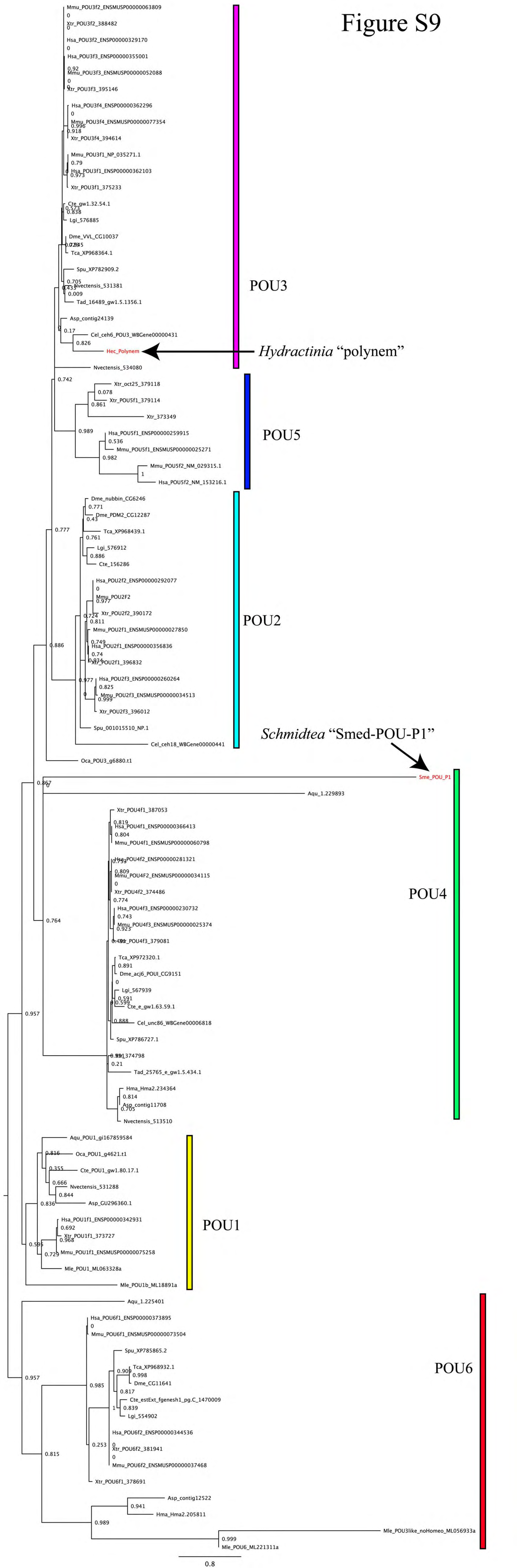

Mmu_POU3f2_ENSMUSP00000063809
Xtr_POU3f2_388482
Hsa_POU3f2_ENSP00000329170
Hsa_POU3f3_ENSP00000355001
Mmu_POU3f3_ENSMUSP00000052088
Xtr_POU3f3_395146
Hsa_POU3f4_ENSP00000362296
Mmu_POU3f4_ENSMUSP00000077354
Xtr_POU3f4_394614
Mmu_POU3f1_NP_035271.1
Hsa_POU3f1_ENSP00000362103
Xtr_POU3f1_375233
Cte_gw1.32.54.1
Lgi_576885
Dme_VVL_CG10037
Tca_XP968364.1
Spu_XP782909.2
Nvectensis_531381
Tad_16489_gw1.5.1356.1
Asp_contig24139
Cel_ceh6_POU3_WBGene00000431
Hec_Polynem
Nvectensis_534080
POU3
Hydractinia "polynem"
Xtr_oct25_379118
Xtr_POU5f1_379114
Xtr_373349
Hsa_POU5f1_ENSP00000259915
Mmu_POU5f1_ENSMUSP00000025271
Mmu_POU5f2_NM_029315.1
Hsa_POU5f2_NM_153216.1
POU5
Dme_nubbin_CG6246
Dme_PDM2_CG12287
Tca_XP968439.1
Lgi_576912
Cte_156286
Hsa_POU2f2_ENSP00000292077
Mmu_POU2F2
Xtr_POU2f2_390172
Mmu_POU2f1_ENSMUSP00000027850
Hsa_POU2f1_ENSP00000356836
Xtr_POU2f1_396832
Hsa_POU2f3_ENSP00000260264
Mmu_POU2f3_ENSMUSP00000034513
Xtr_POU2f3_396012
Spu_001015510_NP.1
Cel_ceh18_WBGene00000441
POU2
Oca_POU3_g6880.t1
Schmidtea "Smed-POU-P1"
Sme_POU_P1
Aqu_1.229893
Xtr_POU4f1_387053
Hsa_POU4f1_ENSP00000366413
Mmu_POU4f1_ENSMUSP00000060798
Hsa_POU4f2_ENSP00000281321
Mmu_POU4F2_ENSMUSP00000034115
Xtr_POU4f2_374486
Hsa_POU4f3_ENSP00000230732
Mmu_POU4f3_ENSMUSP00000025374
Xtr_POU4f3_379081
Tca_XP972320.1
Dme_acj6_POUI_CG9151
Lgi_567939
Cte_e_gw1.63.59.1
Cel_unc86_WBGene00006818
Spu_XP786727.1
Tad_25765_e_gw1.5.434.1
Hma_Hma2.234364
Asp_contig11708
Nvectensis_513510
POU4
Aqu_POU1_gi167859584
Oca_POU1_g4621.t1
Cte_POU1_gw1.80.17.1
Nvectensis_531288
Asp_GU296360.1
Hsa_POU1f1_ENSP00000342931
Xtr_POU1f1_373727
Mmu_POU1f1_ENSMUSP00000075258
Mle_POU1_ML063328a
Mle_POU1b_ML18891a
POU1
Aqu_1.225401
Hsa_POU6f1_ENSP00000373895
Mmu_POU6f1_ENSMUSP00000073504
Spu_XP785865.2
Tca_XP968932.1
Dme_CG11641
Cte_estExt_fgenesh1_pg.C_1470009
Lgi_554902
Hsa_POU6f2_ENSP00000344536
Xtr_POU6f2_381941
Mmu_POU6f2_ENSMUSP00000037468
Xtr_POU6f1_378691
Asp_contig12522
Hma_Hma2.205811
Mle_POU3like_noHomeo_ML056933a
Mle_POU6_ML221311a
POU6
0.8

Figure S10

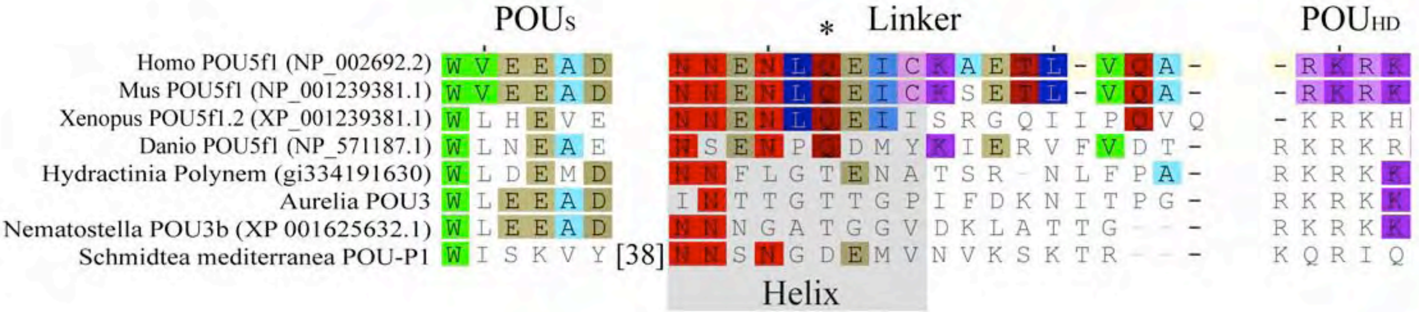
POUs
Linker
*
POUHD
Homo POU5f1 (NP_002692.2)
Mus POU5f1 (NP_001239381.1)
Xenopus POU5f1.2 (XP_001239381.1)
Danio POU5f1 (NP_571187.1)
Hydractinia Polynem (gi334191630)
Aurelia POU3
Nematostella POU3b (XP 001625632.1)
Schmidtea mediterranea POU-P1
[38]
Helix

Figure S11

Type I Functional Divergence Test (Model-Free Estimate)

Comparison	Theta MFE	Theta SE	Z-Score	P-value	
POU6/POU1	0.41821	0.137688	3.03737435	0.0024	*
POU6/POU4	0.609626	0.145296	4.19575212	0	*
POU6/POU2	0.29282	0.109519	2.67369132	0.0075	*
POU6/POU5	0.298019	0.153194	1.94536992	0.0517	
POU6/POU3	0.812979	0.170143	4.77821009	0	*
POU1/POU4	0.430545	0.144625	2.97697494	0.0029	*
POU1/POU2	0.396409	0.13756	2.88171707	0.004	*
POU1/POU5	0.427215	0.183285	2.33087814	0.0198	*
POU1/POU3	0.354213	0.154407	2.29402164	0.0218	*
POU4/POU2	0.300788	0.116438	2.58324602	0.0098	*
POU4/POU5	0.391076	0.168963	2.31456591	0.0206	*
POU4/POU3	0.443081	0.152561	2.90428747	0.0037	*
POU2/POU5	0.509143	0.174826	2.91228421	0.0036	*
POU2/POU3	0.673035	0.165991	4.05464754	0.0001	*
POU5/POU3	0.648905	0.205178	3.16264414	0.0016	*

Type I Functional Divergence Test (ML Estimate)

Comparison	Theta ML	Theta SE	Z-Score	P-value	
POU6/POU1	0.073	0.203712	0.35834904	0.7201	
POU6/POU4	0.5528	0.130875	4.22387775	0	*
POU6/POU2	0.1528	0.176961	0.86346709	0.3879	
POU6/POU5	0.2048	0.180098	1.13715866	0.2555	
POU6/POU3	0.6632	0.275669	2.40578375	0.0161	*
POU1/POU4	0.2624	0.160071	1.63927257	0.1012	
POU1/POU2	0.2152	0.196296	1.09630354	0.2729	
POU1/POU5	0.36	0.150008	2.39987201	0.0164	*
POU1/POU3	0.2896	0.310045	0.93405796	0.3503	
POU4/POU2	0.419988	0.144654	2.90339707	0.0037	*
POU4/POU5	0.5488	0.203356	2.69871555	0.007	*
POU4/POU3	0.479081	0.219927	2.17836373	0.0294	*
POU2/POU5	0.2448	0.130771	1.87197467	0.0612	
POU2/POU3	0.396	0.183833	2.15412902	0.0312	*
POU5/POU3	0.4272	0.489297	0.87308935	0.3826	

Type II Functional Divergence Test

Comparison	Theta-II	Theta SE	Z-Score	P-value	
POU6/POU1	-0.0132	0.214308	-0.06159359	0.9509	
POU6/POU4	0.182972	0.172815	1.05877383	0.2897	
POU6/POU2	0.230561	0.184255	1.25131475	0.2108	
POU6/POU5	0.280273	0.224188	1.2501695	0.2112	
POU6/POU3	0.204315	0.182457	1.11979809	0.2628	
POU1/POU4	0.187941	0.100536	1.86939007	0.0616	
POU1/POU2	0.180125	0.104413	1.72512044	0.0845	
POU1/POU5	0.223505	0.147157	1.51882004	0.1288	
POU1/POU3	0.078114	0.101956	0.76615403	0.4436	
POU4/POU2	0.297267	0.084582	3.5145421	0.0004	*
POU4/POU5	0.337462	0.12556	2.6876553	0.0072	*
POU4/POU3	0.229435	0.078226	2.93297625	0.0034	*
POU2/POU5	0.299312	0.124103	2.4118031	0.0159	*
POU2/POU3	0.130576	0.073733	1.77093025	0.0766	
POU5/POU3	0.075543	0.144337	0.52337931	0.6007	
